# Type I interferon promotes the fate of Toll-like receptor 9–stimulated follicular B cells to plasma cell differentiation

**DOI:** 10.1093/pnasnexus/pgae152

**Published:** 2024-04-17

**Authors:** Ryota Higuchi, Kaori Tanaka, Yuichi Saito, Daisuke Murakami, Takashi Nakagawa, Stephen L Nutt, Yasuyuki Ohkawa, Yoshihiro Baba

**Affiliations:** Division of Immunology and Genome Biology, Medical Institute of Bioregulation, Kyushu University, 3-1-1 Maidashi, Higashi-ku, Fukuoka 812-8582, Japan; Department of Otorhinolaryngology, Graduate School of Medical Sciences, Kyushu University, 3-1-1 Maidashi, Higashi-ku, Fukuoka 812-8582, Japan; Division of Transcriptomics, Medical Institute of Bioregulation, Kyushu University, 3-1-1 Maidashi, Higashi-ku, Fukuoka 812-8582, Japan; Department of Otorhinolaryngology, Graduate School of Medical Sciences, Kyushu University, 3-1-1 Maidashi, Higashi-ku, Fukuoka 812-8582, Japan; Department of Otorhinolaryngology, Graduate School of Medical Sciences, Kyushu University, 3-1-1 Maidashi, Higashi-ku, Fukuoka 812-8582, Japan; Department of Otorhinolaryngology, Graduate School of Medical Sciences, Kyushu University, 3-1-1 Maidashi, Higashi-ku, Fukuoka 812-8582, Japan; The Walter and Eliza Hall Institute of Medical Research, Parkville, VIC 3050, Australia; Department of Medical Biology, The University of Melbourne, Parkville, VIC 3010, Australia; Division of Transcriptomics, Medical Institute of Bioregulation, Kyushu University, 3-1-1 Maidashi, Higashi-ku, Fukuoka 812-8582, Japan; Division of Immunology and Genome Biology, Medical Institute of Bioregulation, Kyushu University, 3-1-1 Maidashi, Higashi-ku, Fukuoka 812-8582, Japan

**Keywords:** B cells, plasma cells, TLR9, type 1 IFN, IRF4

## Abstract

The activation and differentiation of B cells into plasma cells (PCs) play critical roles in the immune response to infections and autoimmune diseases. Toll-like receptor 9 (TLR9) responds to bacterial and viral DNA containing unmethylated CpG motifs and triggers immune responses in B cells; however, abnormal recognition of self-DNA by TLR9 can cause autoimmune diseases. When stimulated with TLR9 agonists, follicular (FO) B cells, a subset of B cells residing in the FO regions of secondary lymphoid organs, exhibit a propensity for activation but fail to give rise to PCs. The factors that enable the transition of TLR9-activated FO B cells from activation to differentiation into PCs remain unclear. In this study, we show that type I interferon-alpha (IFNα) signaling causes FO B cells activated by CpG stimulation to differentiate into PCs. Although CpG stimulation alone only temporarily increased interferon regulatory factor 4 (IRF4) expression in FO B cells, co-stimulation with both CpG and IFNα enhanced and maintained high IRF4 expression levels, ultimately enabling the cells to differentiate into PCs. Overexpression of IRF4 in FO B cells results in CpG-induced PC transition without IFN signaling. Furthermore, co-stimulation of TLR9 and IFNα receptors significantly enhanced mammalian target of rapamycin (mTOR) signaling, which regulates IRF4 expression and PC generation. These findings suggest that IFNα may play a key role in promoting the fate of PC differentiation in FO B cells activated by TLR9 stimulation.

**Significance Statement**

B cells are essential for humoral immunity since they differentiate into plasma cells (PCs). Toll-like receptor 9 (TLR9) detects unmethylated CpG motifs in pathogenic or self-DNA to activate B-cell immune responses. Paradoxically, when follicular (FO) B cells are stimulated with TLR9 agonists alone, they become activated but do not differentiate into PCs, although the underlying mechanism is unknown. In this study, we found that interferon-alpha (IFNα) triggers the differentiation of TLR9-stimulated FO B cells into PCs. After CpG stimulation, FO B cells expressed interferon regulatory factor 4 (IRF4) transiently, but co-stimulation with CpG and IFNα resulted in sustained high IRF4 expression levels through the mammalian target of rapamycin (mTOR) signaling pathway, highlighting the pivotal role of IFNα in PC differentiation of TLR9-activated FO B cells.

## Introduction

Regulation of the immune response to infectious and autoimmune diseases depends on the complex interactions of various immune cell populations, each with its own receptors and signaling pathways. Among these, B cells play a central role in humoral immunity as they undergo activation and differentiation into antibody-secreting plasma cells (PCs) (1, 2). Toll-like receptor 9 (TLR9), a pattern recognition receptor, has been extensively studied for its role in B-cell activation, particularly in response to pathogenic nucleic acids. TLR9 is a critical nucleic acid sensor that responds to bacterial and viral DNA, specifically double-stranded DNA containing unmethylated CpG motifs (3). Upon detecting these motifs, TLR9 triggers a signaling cascade that activates B-cell immune responses, allowing the host to effectively combat pathogens (4, 5). Under typical conditions, nucleic acids from the body, including those from dead cells, are usually degraded by enzymes such as DNase and are not detected by TLR9 (6). While TLR9 may recognize self-DNA for anti-inflammatory effects during infection and for homeostasis (7, 8), TLR9 stimulation for some reason causes the production of antinuclear antibodies in the pathology of autoimmune diseases, such as systemic lupus erythematosus (SLE) (9–13). However, the influence of TLR9 on B-cell differentiation remains unclear.

Remarkably, follicular (FO) B cells, a major subset of B cells, have been observed to exhibit unique responses to TLR9 stimulation. When stimulated with TLR9 agonists, FO B cells proliferate vigorously and show activation but fail to give rise to PCs (14–17). This observation raises fundamental questions regarding the factors that enable FO B cells to transition from activation to differentiation into PCs within the immune microenvironment. In this context, it is puzzling that many B cells activated via TLR9 during infections and autoimmune diseases differentiate into PCs, leading to effective or pathogenic humoral immune responses. Notably, in both infections and autoimmunity, the levels of type I interferon-alpha (IFNα) are elevated (18, 19). In clinical relevance, it has been reported that the pathogenesis of SLE is ameliorated by antibodies to IFNα, and patients with hepatitis or malignancy develop SLE when administered IFNα (20). In mouse models of autoimmune disease, the acceleration of disease progression is not only attributable to TLR9 but also involves IFNα (10, 12, 21). Dnase1L3 knockout mice show increased production of extrafollicular PCs and develop SLE; however, when these mice were crossed with IFNα receptor (IFNαR)-knockout mice, their symptoms improved, suggesting a complex interplay involving TLR9, IFNα, and PC differentiation in the context of autoimmune diseases (22). These observations prompted us to speculate that IFNα may play an essential role in influencing the fate of FO B cells.

In this study, we showed that FO B cells undergo differentiation into PCs when exposed to the synergistic effects of TLR9 activation and IFNα. Mechanistically, stimulation with CpG alone transiently increased interferon regulatory factor 4 (IRF4) expression, whereas the addition of IFNα enhanced and maintained high IRF4 expression levels. These may play a critical role in the differentiation of PCs from FO B cells because forcing IRF4 expression in TLR9-activated FO B cells causes them to differentiate into PCs. Furthermore, we showed that co-stimulation with CpG and IFNα facilitated mammalian target of rapamycin (mTOR) signaling, which is essential for IRF4 expression and PC transition. Thus, these findings suggest the key role of IFNα in promoting the fate of PC differentiation in FO B cells activated by TLR9 stimulation.

## Results

### IFNα allows TLR9-stimulated FO B cells to differentiate into PCs

**Fig. 1.**
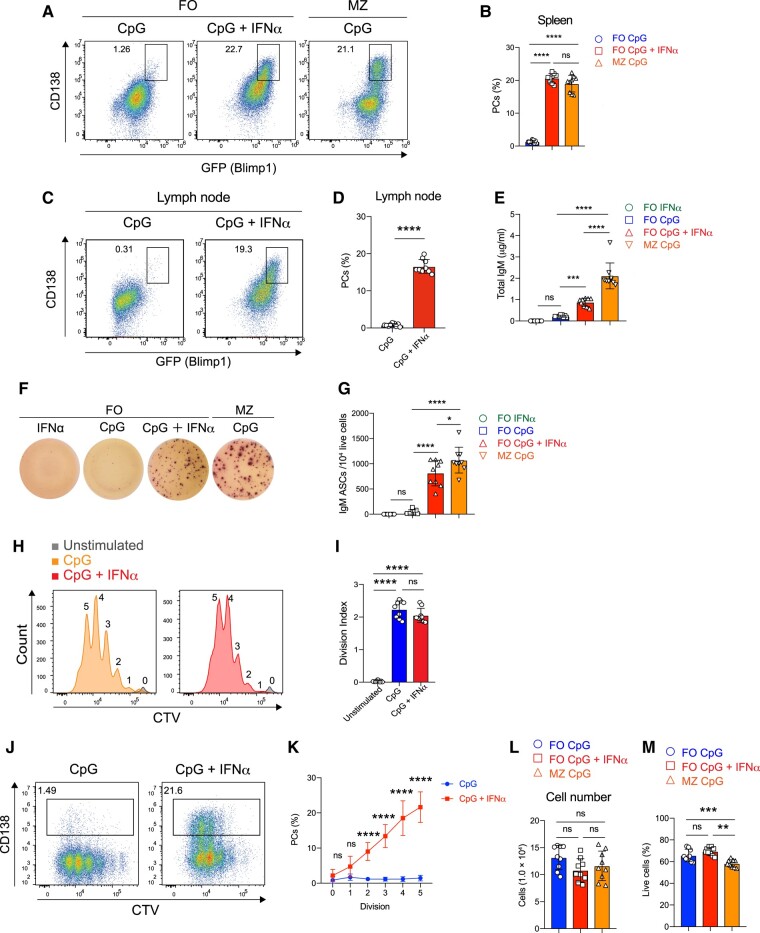
IFNα allows TLR9-stimulated FO B cells to differentiate into PCs. A) Representative flow cytometry plots of B cells harvested from the spleen of *Prdm1*^gfp/+^ (Blimp1-GFP) mice 3 days after culture with CpG (1 μg/mL) or CpG (1 μg/mL) plus IFNα (0.1 μg/mL). The percentages of Blimp1^+^CD138^+^ cells (PCs) are shown. B) The percentages of PCs in (A). C) Representative flow cytometry plots of FO B cells harvested from the lymph node of Blimp1-GFP mice 3 days after culture with CpG or CpG plus IFNα. D) The percentages of PCs in (C). E) The IgM antibody concentration in culture supernatants of B cells harvested from the spleen of Blimp1-GFP mice 3 days after culture with IFNα, CpG, or CpG plus IFNα was measured by ELISA. F) Representative wells of total IgM ELISPots on cells 3 days after stimulation of FO B cells from the spleen of Blimp1-GFP mice with IFNα, CpG, or CpG plus IFNα. G) The number of IgM antibody-secreted cells (ASCs) in (F). H) Representative flow cytometry plots of CTV—stained FO B cells unstimulated (Unstimulated) or stimulated for 3 days with CpG or CpG plus IFNα. I) Division index in (H). J) Representative flow cytometry plots of (H). The percentages of CD138^+^ PCs are shown. K) The percentages of CD138^+^ PCs in (J). L) Cell number of B cells harvested from the spleen of Blimp1-GFP mice 3 days after culture with CpG or CpG plus IFNα. The culture was started at 4.0 × 10^4^ cells per 96-well-round bottom plate. M) The percentages of live cells (propidium iodide–negative cells) in (L). The data are pooled from three independent experiments performed in triplicates (B, D, E, G, I, and K–M). The data are presented as mean ± SD. ns, not significant. *P < 0.05, **P < 0.01, ***P < 0.005, and ****P < 0.001 by one-way ANOVA (B, E, G, I, L, and M) or Student's t test (D) or two-way ANOVA (K).

To assess the influence of IFNα on TLR9-activated B cells for PC differentiation, we stimulated splenic B cells from *Prdm1*^gfp/+^ (Blimp1-GFP) mice or wild-type (WT) mice with the TLR9 agonist CpG and IFNα. As reported previously, marginal zone (MZ) B cells differentiated into PCs when stimulated with CpG alone, whereas FO B cells did not, even with higher concentrations of CpG (Figs. 1A, S1, and S2A, B) (14, 23, 24). However, we found that when IFNα was added, splenic FO B cells differentiated into Blimp1^+^CD138^+^PCs upon CpG stimulation (Fig. 1A and B). Essentially, the same results were obtained with B cells of the lymph nodes, which contain FO B cells but not MZ B cells (Fig. 1C and D). Furthermore, differentiation of CD138^+^ PCs from splenic FO B cells of WT mice was observed in the presence of >20 pg/mL IFNα, with a dose-dependent increase in efficiency (Fig. S2C and D). Enzyme-linked immunosorbent assay (ELISA) and enzyme-linked immunospot (ELISpot) analyses also confirmed that FO B cell-derived PCs produced antibodies (Fig. 1E–G). Furthermore, the frequency of proliferation and survival of FO B cells did not change when they were stimulated with CpG alone or co-stimulated with CpG and IFNα (Fig. 1H–M). Thus, these findings indicate that when FO B cells are exposed to CpG stimulation in the presence of IFNα, they undergo differentiation into PCs. This phenomenon appears to be common for type I IFNs as demonstrated by the observation of similar PC differentiation in IFNβ and universal type I IFN but not in IFNγ (Fig. S2E and F).

### Increased TLR9 expression does not promote PC differentiation of FO B cells

**Fig. 2.**
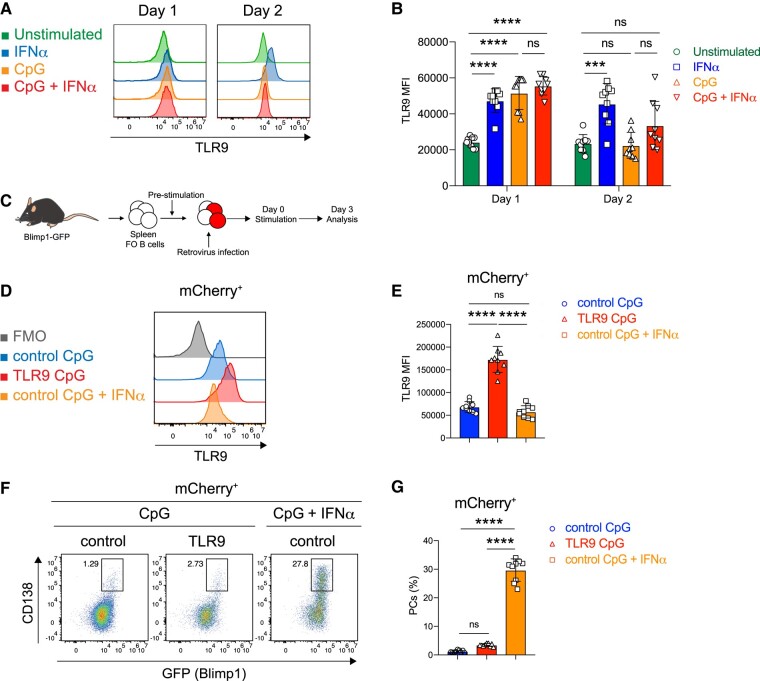
Increased TLR9 expression does not determine PC differentiation of FO B cells. A) Representative histogram of TLR9 in unstimulated FO B cells (Unstimulated) or FO B cells was stimulated with IFNα (0.1 μg/mL), CpG (1 μg/mL), or CpG (1 μg/mL) plus IFNα (0.1 μg/mL) for 1 or 2 days. B) Mean fluorescence intensity (MFI) of TLR9 in (A). C) The scheme of the experiment of FO B cells harvested from the spleen of Blimp1-GFP mice transduced with mCherry (control) or TLR9 is shown. D) Representative histogram of TLR9 in FO B cells after retroviral transduction stimulated with CpG or CpG plus IFNα. Transduced cells were identified by mCherry fluorescence. E) MFI of TLR9 expression of FO B cells in (D). F) Flow cytometry analysis of PC differentiation after retroviral transduction stimulated with CpG (1 μg/mL) or CpG (1 μg/mL) plus IFNα (0.1 μg/mL). Transduced cells were identified by mCherry fluorescence. G) The percentages of Blimp1^+^CD138^+^ cells (PCs) in (F). The data are pooled from three independent experiments performed in triplicates (B, E, and G). The data are presented as mean ± SD. ns, not significant. ***P < 0.005, and ****P < 0.001 by two-way ANOVA (B), one-way ANOVA (E and G).

IFNα stimulation of both human and mouse B cells has been reported to increase *TLR9* expression (25, 26). Therefore, we hypothesized that stimulation of FO B cells with IFNα may up-regulate TLR9 expression and facilitate more PC differentiation. To test this hypothesis, we first determined when FO B cells began to differentiate into PCs under CpG plus IFNα stimulation (Fig. S3), and detected PC differentiation on day 3. We then examined TLR9 expression in FO B cells for up to 2 days after stimulation with CpG, IFNα, or CpG plus IFNα, which corresponded to the time before transition to PCs. Flow cytometry showed that on day 1, all stimuli up-regulated TLR9 expression to the same extent (Fig. 2A and B). On day 2, TLR9 expression remained high only with IFNα stimulation but decreased to unstimulated levels with CpG or CpG and IFNα stimulation (Fig. 2A and B). To more closely and directly examine the effect of TLR9 expression on the CpG-driven PC differentiation of FO B cells, we overexpressed TLR9 in FO B cells (Fig. 2C). Although FO B cells with forced TLR9 expression showed significantly higher TLR9 levels than stimulated FO B cells (Fig. 2D and E), they did not differentiate into PCs upon exposure to CpG alone (Fig. 2F and G). These data suggest that the expression levels of TLR9 do not promote the differentiation of FO B cells into PCs.

### Co-stimulation of CpG and IFNα promotes and maintains high expression of IRF4

**Fig. 3.**
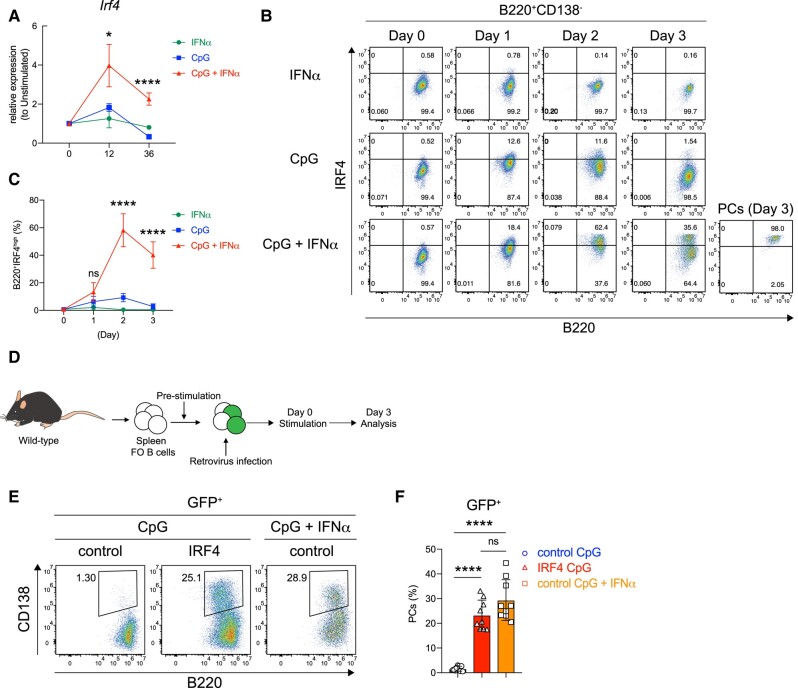
Co-stimulation of CpG and IFNα promotes and maintains high expression of IRF4. A) Real-time PCR analysis of *Irf4* after FO B cells was stimulated with IFNα (0.1 μg/mL), CpG (1 μg/mL), or CpG (1 μg/mL) plus IFNα (0.1 μg/mL). B) Representative flow cytometry plots of FO B cells harvested from the spleen of wild-type mice 1–3 days after culture with IFNα, CpG, or CpG plus IFNα. The percentages of each fraction are shown. C) The percentage of B220^+^IRF4^high^ cells in (A). D) The scheme of the experiment of FO B cells transduced with GFP (control) or IRF4 is shown. E) Flow cytometry analysis of PC differentiation after retroviral transduction. Transduced cells were identified by GFP fluorescence. F) The percentages of PCs in (E). The data are representative of three independent experiments performed in triplicates (A) or pooled from three independent experiments performed in triplicates (C and F). The data are presented as mean ± SD. ns, not significant. *P < 0.05, and ****P < 0.001 by two-way ANOVA (A and C) or one-way ANOVA (F).

The process of PC differentiation occurs under strict control of various transcription factors, including those that suppress B cell-associated transcripts, such as *Pax5*, while activating genes that facilitate PC differentiation, such as *Irf4* and *Prdm1* (2, 27–31). IRF4 is crucial for initiating PC differentiation, and its concentration level is directly linked to this process (32–34). To understand the mechanism underlying PC differentiation by stimulation of FO B cells with CpG and IFNα, we next examined IRF4 protein and mRNA expression levels in B cells over time. Quantitative polymerase chain reaction analysis revealed that the levels of the *Irf4* transcript increased with CpG stimulation but were significantly enhanced with concurrent stimulation of IFNα (Fig. 3A). Flow cytometry showed that IRF4 protein expression in FO B cells transiently increased after stimulation with CpG alone but was not maintained or decreased (Fig. 3B and C). In contrast, stimulation with CpG and IFNα markedly increased and maintained IRF4 levels. These findings strongly suggest that the effects of IFNα in TLR9-activated FO B cells may involve increasing IRF4 expression to the levels required for PC differentiation. We then hypothesized that enhancing and maintaining IRF4 concentrations could induce PC differentiation with CpG stimulation alone. To test this, IRF4 was retrovirally overexpressed in FO B cells stimulated with CpG alone (Figs. 3D and S4A). We observed that enhancement of IRF4 expression enabled FO B cells to give rise to PCs after CpG stimulation, in comparison with mock cells stimulated with CpG and IFNα (Fig. 3E and F). Additionally, the forced expression of IRF4 in CpG + IFNα-stimulated FO B cells further enhanced PC differentiation (Fig. S4B–E). These data suggest that IFNα is a key to promoting and maintaining high levels of IRF4 expression in TLR9-activated FO B cells, allowing PC generation.

### Unique gene expression profile in FO B cells stimulated with CpG and IFNα

**Fig. 4.**
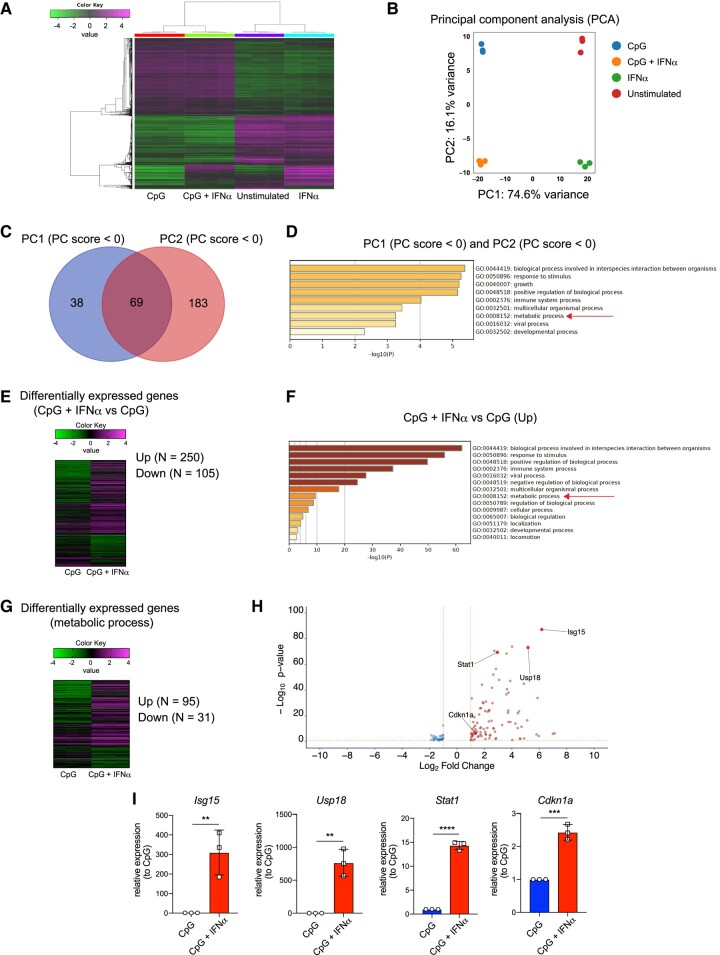
Unique gene expression profile in FO B cells was stimulated with CpG and IFNα. A) RNA-seq heat map of DEGs from sorted splenic FO B cells unstimulated (Unstimulated) or stimulated with IFNα (0.1 μg/mL), CpG (1 μg/mL), or CpG (1 μg/mL) plus IFNα (0.1 μg/mL) for 12 h. The 1,000 most variable genes are shown. B) PCA of (A). C) Venn diagram of PC1 (PC score <0) and PC2 (PC score <0) in (B). D) The top-level GO biological processes of both PC1 (PC score <0) and PC2 (PC score <0) in (C) by Metascape. E) Heat map of DEGs of CpG plus IFNα vs. CpG. False discovery rate (FDR) cutoff <0.1, fold change >2. F) The top-level up-regulated-GO biological processes of (E) by Metascape. G) Heat map of DEGs of CpG plus IFNα vs. CpG. FDR cutoff <0.1, fold change >2. H) Volcano plot of (G) *P*-value cutoff <0.1, log_2_ fold change >1. I) Real-time PCR analysis of *Isg15*, *Usp18*, *Stat1*, and *Cdkn1a* after FO B cells were stimulated for 12 h with CpG or CpG plus IFNα. The data are representative of two independent experiments performed in triplicates (I). The data are presented as mean ± SD. ns, not significant. **P < 0.01, ***P < 0.005, and ****P < 0.001 by Student's t test (I).

To identify the pathways that alter IRF4 expression in FO B cells, we sorted FO B cells, stimulated them with CpG, IFNα, and CpG plus IFNα for 12 h prior to the onset of PC differentiation, and conducted an RNA-Seq analysis (Fig. 4A). FO B cells stimulated with IFNα, CpG, and CpG plus IFNα showed differentially expressed genes (DEGs; Fig. S5A–C). Principal component analysis (PCA) revealed that FO B cells stimulated with CpG and IFNα were clearly distinguished from those stimulated with CpG or IFNα (Fig. 4B). To identify the most important pathways for PC differentiation, we determined which genes were differentially expressed in each sample. Gene ontology (GO) analysis of genes from PCA, in which both PC1 and PC2 had PC score <0, suggests that they contribute significantly to the stimulation of CpG plus IFNα, indicated by the high enrichment of several biological processes (Fig. 4C and D). We presented a heat map displaying DEGs up-regulated by CpG and CpG + IFNα stimulation (Fig. 4E and Table S1). The GO analysis revealed that the enriched genes were associated with biological events, such as “the response to stimuli,” “metabolic processes,” and “immune system processes” (Fig. 4F). Since the results of both pathway analyses included metabolic processes involved in PC differentiation (16, 25, 35, 36), we confirmed their expression by qPCR analysis for representative genes and focused on them (Fig. 4G–I and Table S2), specifically mTOR.

### mTOR promotes up-regulation of IRF4 and enables FO B cells to undergo PC differentiation

**Fig. 5.**
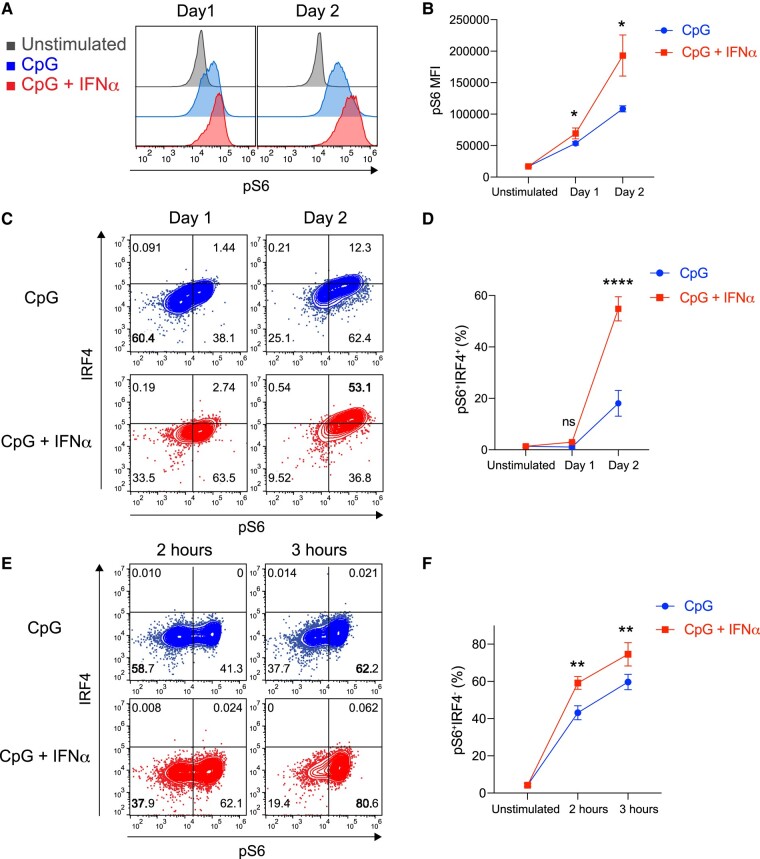
mTORC1 promotes up-regulation of IRF4 and enables FO B cells to PC differentiation. A) Representative histogram of pS6 in FO B cells unstimulated (Unstimulated) or stimulated with CpG (1 μg/mL) or CpG (1 μg/mL) plus IFNα (0.1 μg/mL). B) MFI of pS6 in (A). C) Representative flow cytometry plots of FO B cells harvested from the spleen of wild-type mice 1 or 2 days after culture with CpG or CpG plus IFNα. D) The percentages of pS6^+^IRF4^+^ in (C). E) Representative flow cytometry plots of FO B cells harvested from the spleen of wild-type mice 2 or 3 h after culture. The percentages of FO B cells in each fraction are shown. F) The percentages of pS6^+^IRF4^−^ cells in (E). The data are representative of three independent experiments performed in triplicates (B, D, and F). The data are presented as mean ± SD. ns, not significant. *P < 0.05, **P < 0.01, and ****P < 0.001 by Student's t test (B, D, and F).

We hypothesized that IFNα stimulation of FO B cells activated through TLR9 would alter the mTOR pathway, leading to increased expression of IRF4 and subsequent differentiation into PCs. Activated mTOR phosphorylates ribosomal protein S6, which ultimately affects various cellular processes in many cells, including B cells (37–41). We observed that stimulation with CpG plus IFNα resulted in greater enhancement of the phosphorylation of S6 (pS6) than stimulation with CpG alone (Fig. 5A and B). Even when the concentration of CpG was increased, pS6 was not enhanced by stimulation with only CpG (Fig. S6A). Additionally, the percentage of pS6-positive cells that are IRF4 positive was much higher with the addition of IFNα (Fig. 5C and D). This phenomenon was observed even at earlier stimulation times before IRF4 was detected (Fig. 5E and F). These findings suggest that IFNα may promote signaling to S6 downstream mTOR in CpG-activated FO B cells.

### Inhibition of mTORC1 prevents differentiation of FO B cells when stimulated with CpG and IFNα

**Fig. 6.**
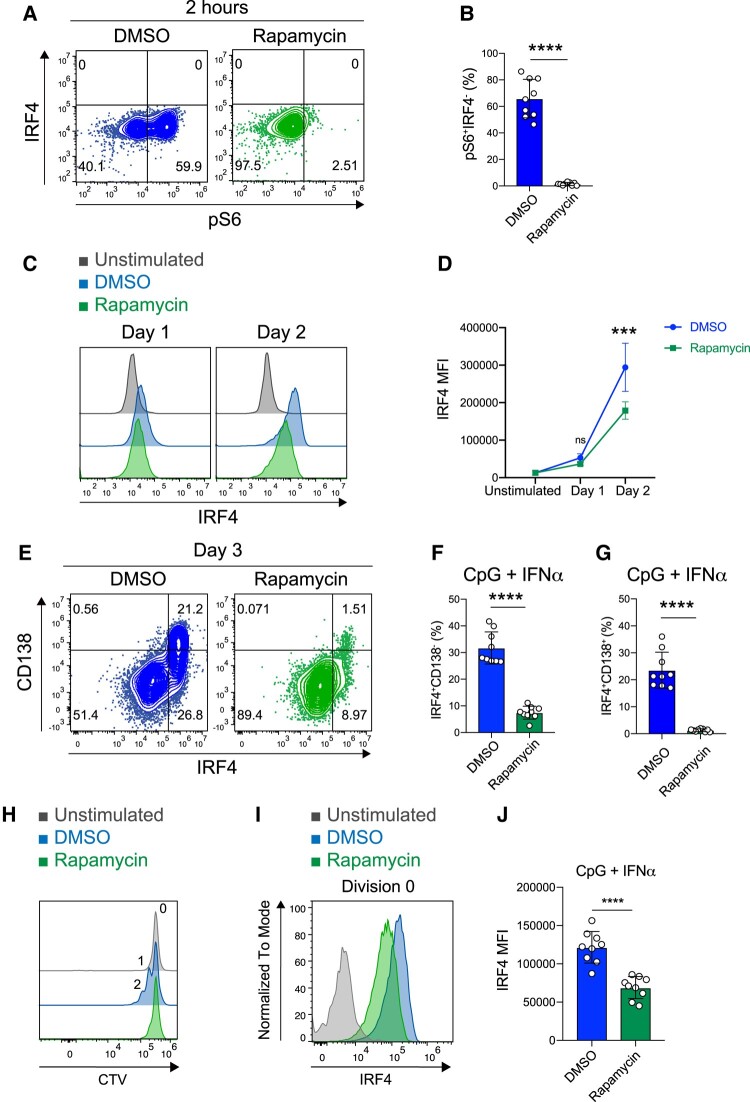
Inhibition of mTORC1 prevents differentiation of FO B cells when stimulated with CpG and IFNα. A) Representative flow cytometry plots of FO B cells were stimulated for 2 hours with CpG (1 μg/mL) plus IFNα (0.1 μg/mL) in the presence of dimethyl sulfoxide (DMSO) or rapamycin (mTORC1 inhibitor). The percentages of B cells in each fraction are shown. B) The percentages of pS6^+^IRF4^−^ cells in (A). C) Representative histogram of IRF4 in unstimulated FO B cells (Unstimulated) or FO B cells was stimulated with CpG plus IFNα in the presence of DMSO or rapamycin. D) MFI of IRF4 in (C). E) Representative flow cytometry plots of FO B cells were stimulated for 3 days with CpG plus IFNα in the presence of DMSO or rapamycin. The percentages of FO B cells in each fraction are shown. F and G) The percentages of IRF4^+^CD138^−^ (F) or IRF4^+^CD138^+^ (G) in (E). H) Representative histogram of the proliferation of splenic FO B cells labeled with CTV and stimulated for 2 days with CpG plus IFNα in the presence of DMSO or rapamycin. I) Representative histogram of IRF4 in division 0 of (H). J) MFI of IRF4 in (I). The data are pooled from three independent experiments performed in triplicates (B, F, G, and J) or representative of three independent experiments performed in triplicates (D). The data are presented as mean ± SD. ns, not significant. ***P < 0.005 and ****P < 0.001 by Student's t test (B, D, F, G, and J).

To determine whether mTORC1 is required for IRF4 expression and PC differentiation after CpG and IFNα stimulation, FO B cells were stimulated with CpG and IFNα in the presence of rapamycin, an inhibitor of the mTORC1 pathway. Two hours after stimulation, rapamycin inhibited pS6 (Fig. 6A and B). In FO B cells stimulated with CpG and IFNα, treatment with rapamycin markedly reduced IRF4 expression (Fig. 6C and D). Further analysis showed that rapamycin inhibited the differentiation of IRF4^+^CD138^+^ PCs (Fig. 6E–G). It should be noted that rapamycin inhibits FO B-cell proliferation after stimulation with CpG and IFNα (Fig. S7A and B). As PC differentiation from FO B cells is associated with cell division, we investigated whether rapamycin inhibits IRF4 expression independently of cell division. We found that CpG + IFNα stimulation leads to increased IRF4 expression in FO B cells even before cell division, and rapamycin can inhibit IRF4 expression in nondivided FO B cells (Fig. 6H–J). We also observed that retroviral overexpression of IRF4 failed to abrogate the inhibitory effect of rapamycin on PC differentiation (Fig. S7C–G). These data suggest that rapamycin inhibits the differentiation of PC from FO B cells by affecting both cell division and IRF4 expression. In addition to mTORC1 signaling, inhibition of phosphatidylinositol-3 kinase (PI3K) and the protein kinase v-akt murine thymoma viral oncogene homolog (Akt) decreased PC differentiation after CpG and IFNα stimulation (Fig. S7H–J). These results suggest that PI3K/Akt/mTOR signaling is required for up-regulation and maintenance of IRF4 and PC differentiation in FO B cells co-stimulated with CpG and IFNα.

## Discussion

While TLR9 is expressed in various mature B-cell populations, including MZ B cells and FO B cells, MZ B cells have been shown to possess the ability to differentiate into PCs upon CpG stimulation. Intriguingly, this capability is not shared by FO B cells, despite expressing TLR9. In this study, we demonstrated that FO B cells can differentiate into PCs upon CpG stimulation in the presence of IFNα. Notably, IFNα sustained high expression levels of IRF4, a key factor in PC differentiation, in FO B cells. Mechanistically, co-stimulation of CpG and IFNα markedly enhanced mTORC1 signaling, a pathway essential for IRF4 expression and PC generation.

TLR9 agonists have been reported to promote B-cell proliferation and may be effective as vaccine adjuvants. Some studies have suggested that TLR9 signaling induces the differentiation of B cells into antibody-secreting cells (42); however, the findings of experiments involving in vitro stimulation of splenic B cells should be interpreted carefully. Consistent with our results, several studies have reported that FO B cells rarely differentiate into PCs upon CpG stimulation in vitro, whereas MZ B cells do (14, 23). Our findings showing that IFNα enables FO B cells to generate PCs have essential implications for the existing understanding of these cells. In situations such as infections or vaccinations, plasmacytoid dendritic cells may be activated via TLR9 to produce IFNα. When a TLR9 ligand and IFNα are combined, they can stimulate FO B cells to differentiate into PCs. This process can be beneficial during acute infections, as it allows for rapid production of antibodies.

In the context of autoimmunity, since TLR9 is involved in self-DNA recognition (7, 8), it is likely that autoreactive FO B cells are stimulated by TLR9 and induce PC differentiation in an elevated IFNα environment. Autoimmune diseases, such as SLE, are associated with elevated levels of type I interferons and increased expression of genes regulated by them in B cells, which contribute to pathogenesis (19). TLR9 activation alone limits the differentiation of FO B cells into PCs, but elevated IFNα levels may alter this fate and promote the differentiation of these B cells into PCs. Therefore, low levels of IFNα in the healthy or uninfected state may prevent harmful FO B cells from differentiating into PCs when stimulated by TLR9.

The process of PC differentiation occurs under the strict control of various transcription factors, including those that suppress B cell-associated transcripts, such as *Pax5*, while activating genes that facilitate PC differentiation, such as *Irf4* and *Prdm1* (2, 27–30). IRF4 plays a crucial role in initiating PC differentiation, and high concentrations of IRF4 can lead to successful differentiation (32–34). Similar to the differentiation outcomes observed in PCs, TLR9 stimulation up-regulates the *Prdm1* gene (14, 23, 24) and Blimp1 protein in MZ B cells but not in FO B cells. However, the addition of IFNα resulted in the up-regulation of Blimp1 expression in FO B cells. Interestingly, our findings indicate that CpG stimulation transiently increased IRF4 expression in FO B cells, but co-stimulation with CpG and IFNα resulted in sustained and stronger expression. Since the accumulation of IRF4 in activated B cells promotes the generation of PCs, TLR9 signaling alone is insufficient to increase IRF4 concentrations in FO B cells. Consistent with this finding, our data indicate that the overexpression of IRF4 causes FO B cells to give rise to PCs upon CpG stimulation alone. These findings suggest that the critical role of IFNα is to generate the necessary levels of IRF4 for PC differentiation.

Although IFNα signals regulate high IRF4 concentration in TLR9-activated FO B cells, IFNα stimulation alone does not induce IRF4 expression. IFNα regulates or synergizes the TLR9 signaling pathway (25, 26). *Irf4* gene expression is controlled by several pathways, such as the nuclear factor kappa B (NF-κB), PI3K, Akt, and mTOR axis (32, 43, 44). In the present study, co-stimulation with TLR9 agonist and IFNα enhanced pS6 expression in FO B cells before increasing IRF4 expression, while treatment with rapamycin, mTORC1 inhibitor, suppressed pS6 and IRF4 expression, resulting in reduced PC differentiation. Previous studies have shown that the mTOR pathway is crucial for PC differentiation (16, 24, 35, 36, 37). In addition, the inactivation of Tsc1, which suppresses mTORC1 activity, enables FO B cells to differentiate into PCs upon stimulation with CpG alone (24). These findings suggest that activation of the mTORC1 pathway may be crucial for PC differentiation from TLR9-activated FO B cells. Furthermore, the mTORC1 pathway is involved in IRF4 expression in several cell types, including TCR-stimulated CD8^+^ T cells (45) and LPS-stimulated B cells (43). Thus, although the exact points of intersection remain unclear, the engagement of both TLR9 and IFNα receptors enhances the mTOR pathway and subsequent sustained IRF4 expression, which likely plays a role in the transition of FO B cells to PCs. However, we do not exclude the possibility that IFNα provides a unique signal that is lacking in the downstream signaling pathway of TLR9 for PC differentiation. Future studies will help further define the signaling pathway(s) leading to PC transition from TLR9-activated FO B cells.

## Materials and methods

### Mice

C57BL/6 mice were purchased from CLEA Japan. *Prdm1*^gfp/+^ mice (46) have been described previously. Mice were bred and maintained under specific-pathogen-free conditions and used at 7 to 12 weeks of age. All studies and procedures were approved by the Animal Experiment Committee of Kyushu University. All animal experiments were conducted in accordance with the ARRIVE guidelines and the ethical guidelines of Kyushu University.

### Antibodies

For flow cytometry or cell sorting, single-cell suspensions prepared from spleen or lymph nodes, or cultured B cells were stained with the following biotin- or fluorochrome-conjugated antibodies purchased from BioLegend, BD Bioscience, eBioscience, or Cell Signaling Technology: biotin-conjugated anti-CD23 (B3B4); allophycocyanin (APC)-conjugated anti-streptavidin, anti-CD138 (281-2), anti-CD93 (AA4.1), and Alexa647-conjugated anti-IRF4 (3E4); brilliant violet (BV421)-conjugated anti-CD138 (281-2); phycoerythrin (PE)-conjugated anti-CD19 (6D5), anti-CD23 (B3B4), anti-IRF4 (3E4), anti-TLR9 (J15A7), phospho-S6 (pS6) ribosomal protein (Ser235/236) (D57.2.2E); PE-cyanine 7 (PE-Cy7)-conjugated anti-B220 (RA3-6B2), anti-CD21/35 (7E9), fluorescein isothiocyanate (FITC)-conjugated anti-B220 (RA3-6B2), and APC-Cy7-conjugated anti-CD19 (6D5) were used.

### Flow cytometry

Tissues were disrupted by passing through a nylon mesh (Kyoshin Ricoh). After red blood cell lysis with ammonium chloride potassium buffer, cells were incubated with an anti-CD16/CD32 (2.4G2; BD Pharmingen) to reduce nonspecific labeling of the cells before staining. Single cells were stained with fluorophore-labeled antibodies. For intracellular staining, splenocytes were fixed and permeabilized with Intracellular Fixation and Permeabilization Buffer set (eBioscience) or Foxp3 Staining Buffer Set (eBioscience) before intracellular staining with PE-conjugated anti-TLR9, anti-IRF4, pS6, Alexa647-conjugated anti-IRF4, and then analyzed on Cytoflex (Beckman Coulter). The data were acquired on a Cytoflex (Beckman Coulter) and analyzed with FlowJo software (Tree Star).

### Sorting and isolation of B cells

Cell sorting was done on a FACS Melody (BD Biosciences). For isolation of B220^hi^CD19^+^AA4.1^−^CD23^+^CD21^mid^ FO B cells and B220^hi^ CD19^+^AA4.1^−^CD23^−^CD21^hi^ MZ B cells, splenocytes were stained with FITC-anti-B220, APC-Cy7-anti-CD19, APC-anti-CD93, PE-anti-CD23, and PE-Cy7-anti-CD21/35.

For B-cell isolation, splenic B cells were purified by negative selection of CD43^+^ cells with anti-CD43 magnetic beads (Miltenyi Biotec). Following the negative selection of CD43^−^ B cells, for CD23^+^ B-cell isolation, CD43^−^ B cells were purified by the positive selection of CD23^+^ cells Streptavidin magnetic beads (Miltenyi Biotec) after being stained with biotin-anti-CD23. The enriched B-cell population was >95% positive for CD19 staining.

### Cell culture and stimulation

For B-cell stimulation assays, purified B cells (2 × 10^5^ cells/mL) were cultured in Rosewell Park Memorial Institute (RPMI) 1640 medium supplemented with 10% (vol/vol) fetal calf serum (FCS), β-mercaptoethanol, L-glutamine, 4-(2-hydroxyethyl)-1-piperazine ethanesulfonic acid (HEPES), non-essential amino acids (NEAA), L-sodium pyruvate solution, and penicillin–streptomycin. B cells were stimulated with 1 μg/mL of CpG oligodeoxynucleotides (ODN1826; InvivoGen) and 0.1 μg/mL of IFNα (BioLegend) or 0.1 μg/mL of universal type I IFN (BioLegend) or 0.1 μg/mL of IFNβ (BioLegend) or 0.05 μg/mL of IFNγ (BioLegend) for 3 days in 48- or 96-well plates at 37 °C. Small-molecule inhibitors of mTORC1 (Rapamycin; MedChemExpress; 5 nM), PI3K (LY294002; EMD Millipore; 5 μM), and Akt (API-1; Calbiochem; 500 nM) were added to the cells at the time of activation.

### Proliferation assay

Isolated B cells were labeled with 20 μM Cell Trace Violet (CTV; Invitrogen) for 5 min at room temperature. The cells were stimulated with CpG and IFNα for 3 days at 37 °C. Cells were stained with zombie NIR Fixable Viability Kit (BioLegend) and then the percentages of viable-and CTV-diluted B cells were assessed by Cytoflex (Beckman Coulter). The Division Index is the average number of cell divisions undergone by the cells of the original population (47). The data were analyzed with FlowJo software (Tree Star).

### ELISA and ELISpot assay

Total immunoglobulin M (IgM) was measured by ELISA with a plate coated with 0.5 μg/mL anti-IgM and then detected with horseradish peroxidase–conjugated anti-goat IgM Abs (Southern Biotech). ELISpot assay plates (Millipore MSHAN4B50) were coated with capture goat anti-IgM antibody (Southern Biotech 1021-01) at 0.5 μg/mL and blocked with complete growth media (RPMI, 10% FCS, β-mercaptoethanol, L-glutamine, HEPES, NEAA, L-sodium pyruvate solution, and penicillin–streptomycin). About 1 × 10^4^ stimulated FO B cells were plated on the top wells and serially triple-diluted and incubated overnight. Biotinylated goat anti-IgM AP (Southern Biotech 1020-04) capture antibody was used at 0.1 μg/mL. Spots were developed using 5-bromo-4-chloro-3-indolyl-phospate (BCIP)/nitro blue tetrazolium (NBT) liquid substrate.

### Retroviral transduction

To generate a target gene retroviral expression vector, a cDNA corresponding to a target gene obtained from mouse splenocytes by PCR amplification was cloned into the pMY-IRES-mCherry or pMY-IRES-GFP retroviral vector. The target genes are TLR9 or IRF4. The resulting retroviral vector (pMY-IRES-TLR9-mCherry or pMY-IRF4-HA-IRES-GFP) and mCherry or GFP-alone control vector (pMY-IRES-mCherry or pMY-IRES-GFP) were transfected into PLAT-E cells with FuGENE HD (Roche Diagnostics). At 24 h posttransfection, the medium was changed, and the cells were cultured for an additional 72 h. To express these genes in FO B cells in vitro, splenic FO B cells were purified from C57BL6 or *Prdm1*^gfp/+^ mice and then cultured with CpG (1 μg/mL) or CpG (1 μg/mL) plus IFNα (0.1 μg/mL) for 24 h. Cells underwent “spin infection” for 2 hours at 25 °C (800 g) after virus supernatant and polybrene (6 μg/mL) were added. After spin infection, cells were washed with culture medium and cultured for an additional 3 days with CpG or CpG plus IFNα. Rapamycin was added to the cells after spin infection.

### Quantitative RT-PCR analysis

RNA was isolated and purified using the RNeasy kit (Qiagen) from FO B cells. cDNA was generated using the ReverTra Ace qPCR RT Master Mix (TOYOBO). Real-time PCR was performed on a LightCycler 96 (Roche) using Thunderbird SYBR qPCR mix (TOYOBO). The expression of target genes was normalized with 18S rRNA. The following primer pairs were used: *18S rRNA*, sense 5′-ATGGCCGTTCTTAGTTGGTG-3′ and antisense 5′-CGGACATCTAAGGGCATCAC-3′: *Irf4*, sense 5′-ACAGCTCATGTGGAACCTCTG12 3′ and antisense 5′-TCAGGTAACTCGTAGCCCCT-3′: *Isg15*, sense 5′-TGGCCTGGGACCTAAAGGTG-3′ and antisense 5′-CTGGAAAGCCGGCACACCAA-3′: *Usp18*, sense 5′-GGATAACAGTGCCTCGGAGTG-3′ and antisense 5′-TCTGCAGGCACTGAACGAGC-3′: *Stat1*, sense 5′-AAAGCAAGACTGGGAGCACG-3′ and antisense 5′-GGAGATTACGCTTGCTTTTCCG-3′: *Cdkn1a*, sense 5′-GTGGCCTTGTCGCTGTCTTG-3′ and antisense 5′-CGCTTGGAGTGATAGAAATCTG-3′.

### BRB sequencing

RNA was isolated and purified using the RNeasy kit (Qiagen) from unstimulated FO B cells or FO B cells stimulated with IFNα, CpG, and CpG + IFNα for 12 h FO B cells after sorted on an FACS Melody (BD Biosciences). For library preparation, Bulk RNA barcording (BRB) sequencing (48) was performed with the following some modifications. Barcoded dT primer (5′-GCCGGTAATACGACTCACTATAGGGAGTTCTACAGTCCGACGATCNNNNNNNNNNCCCCCCCCCTTTTTTTTTTTTTTTTTTTTTTTTV −3′; (10)N = UMI, (9)C = cell barcode) was used for reverse transcription. Second-strand synthesis module (NEB, #E6111) was used for double-stranded cDNA synthesis. In-house MEDS-B Tn5 transposase (49, 50) was used for tagmentation, and libraries were amplified by 10 cycles of PCR using Phusion High-Fidelity DNA Polymerase (Thermo Scientific, #M0530) with the following primers: 5′-AATGATACGGCGACCACCGAGATCTACACindexGTTCAGAGTTCTACAGTCCGA-3′, 5′-CAAGCAGAAGACGGCATACGAGATindex GTCTCGTGGGCTCGGAGATGT-3′). A 19-bp barcode read (Read1) and a 81-bp insert read (Read2) were obtained using the Illumina NovaSeq6000. Read1 (barcode read) was extracted by using UMI tools (ver. 1.1.2) with the following command “umi_tools extract -I read1.fastq –read2-in=read2.fastq –bc-pattern=NNNNNNNNNNCCCCCCCCC –read2-stdout.” Trim Galore (ver. 0.6.7) was used to remove adaptor sequence and low-quality sequence and to discard read length below 20 bp, and reads were mapped to the GRCm38 reference using HISAT2 (ver. 2.2.1). FeatureCounts (ver. 2.0.1) was used to obtain the read counts for each gene, and UMI duplication is removed by UMI tools with the following command “umi_tools count –method=unique –per-gene –per-cell –gene-tag=XT.” *Tlr9* reference was then extended by 3 kb to add UTR reads to the counts. Expression data (normalized counts) were then imported into iDEP.951 (http://bioinformatics.sdstate.edu/idep95/), and data transformation was conducted using EdgeR (log2(CPM + c)), as described before (51). Hierarchical clustering was conducted using correlation distance and average linkage. DESeq2 (1.34.0) was used to identify DEGs using |log_2_FC| > 2 and padj < 0.1 as threshold values. Metascape (https://metascape.org/gp/index.html#/main/step1) was used for GO analysis, and GO terms were analyzed for biological processes (52). ggVolcanoR (https://ggvolcanor.erc.monash.edu/) was used to create volcano plot (53).

### Statistical analysis

We performed a statistical evaluation with Prism software (GraphPad). A two-tailed, unpaired Student's t test was applied for the statistical comparison of the two groups. ANOVA test was applied to test the difference in means between two or more unresponsive groups. A *P*-value of <0.05 was considered statistically significant.

## Supplementary Material

pgae152_Supplementary_Data

## Data Availability

All sequencing data generated in this study have been deposited in GEO under accession GSE244442 (https://www.ncbi.nlm.nih.gov/geo/query/acc.cgi?acc=GSE244442).

## References

[1] Cyster JG, Allen CDC. 2019. B cell responses: cell interaction dynamics and decisions. Cell. 177:524–540. 31002794 10.1016/j.cell.2019.03.016 PMC6538279

[2] Nutt SL, Hodgkin PD, Tarlinton DM, Corcoran LM. 2015. The generation of antibody-secreting plasma cells. Nat Rev Immunol. 15:160–171. 25698678 10.1038/nri3795

[3] Kumagai Y, Takeuchi O, Akira S. 2008. TLR9 as a key receptor for the recognition of DNA. Adv Drug Deliv Rev. 60:795–804. 18262306 10.1016/j.addr.2007.12.004

[4] Rawlings DJ, Schwartz MA, Jackson SW, Meyer-Bahlburg A. 2012. Integration of B cell responses through Toll-like receptors and antigen receptors. Nat Rev Immunol. 12:282–294. 22421786 10.1038/nri3190 PMC3437941

[5] O’neill SK, et al 2009. Endocytic sequestration of the B cell antigen receptor and Toll-like receptor 9 in anergic cells. Proc Natl Acad Sci U S A. 106:6262–6267. 19332776 10.1073/pnas.0812922106 PMC2662959

[6] Nagata S, Nagase H, Kawane K, Mukae N, Fukuyama H. 2003. Degradation of chromosomal DNA during apoptosis. Cell Death Differ. 10:108–116. 12655299 10.1038/sj.cdd.4401161

[7] Kumar V . 2021. The trinity of cGAS, TLR9, and ALRs guardians of the cellular galaxy against host-derived self-DNA. Front Immunol. 11:624597. 33643304 10.3389/fimmu.2020.624597 PMC7905024

[8] Dolina JS, et al 2020. TLR9 sensing of self-DNA controls cell-mediated immunity to listeria infection via rapid conversion of conventional CD4+ T cells to treg. Cell Rep. 31:107249. 32268093 10.1016/j.celrep.2020.01.040 PMC8903023

[9] Barrat FJ, et al 2005. Nucleic acids of mammalian origin can act as endogenous ligands for Toll-like receptors and may promote systemic lupus erythematosus. J Exp Med. 202:1131–1139. 16230478 10.1084/jem.20050914 PMC2213213

[10] Sisirak V, et al 2016. Digestion of chromatin in apoptotic cell microparticles prevents autoimmunity. Cell. 166:88–101. 27293190 10.1016/j.cell.2016.05.034 PMC5030815

[11] Wen L, et al 2023. Toll-like receptors 7 and 9 regulate the proliferation and differentiation of B cells in systemic lupus erythematosus. Front Immunol. 14:1093208. 36875095 10.3389/fimmu.2023.1093208 PMC9975558

[12] Marshak-Rothstein A . 2006. Toll-like receptors in systemic autoimmune disease. Nat Rev Immunol. 6:823–835. 17063184 10.1038/nri1957 PMC7097510

[13] Tilstra JS, et al 2020. B cell-intrinsic TLR9 expression is protective in murine lupus. J Clin Invest. 130:3172–3187. 32191633 10.1172/JCI132328 PMC7260024

[14] Genestier L, et al 2007. TLR agonists selectively promote terminal plasma cell differentiation of B cell subsets specialized in thymus-independent responses. J Immunol. 178:7779–7786. 17548615 10.4049/jimmunol.178.12.7779

[15] Baptista BJA, et al 2018. TLR9 signaling suppresses the canonical plasma cell differentiation program in follicular b cells. Front Immunol. 9:2281. 30546358 10.3389/fimmu.2018.02281 PMC6279956

[16] Gaudette BT, Jones DD, Bortnick A, Argon Y, Allman D. 2020. mTORC1 coordinates an immediate unfolded protein response-related transcriptome in activated B cells preceding antibody secretion. Nat Commun. 11:723. 32024827 10.1038/s41467-019-14032-1 PMC7002553

[17] Nündel K, et al 2015. Cell-intrinsic expression of TLR9 in autoreactive B cells constrains BCR/TLR7-dependent responses. J Immunol. 194:2504–2512. 25681333 10.4049/jimmunol.1402425 PMC4382804

[18] McNab F, Mayer-Barber K, Sher A, Wack A, O’Garra A. 2015. Type I interferons in infectious disease. Nat Rev Immunol. 15:87–103. 25614319 10.1038/nri3787 PMC7162685

[19] Weckerle CE, et al 2011. Network analysis of associations between serum interferon-α activity, autoantibodies, and clinical features in systemic lupus erythematosus. Arthritis Rheum. 63:1044–1053. 21162028 10.1002/art.30187 PMC3068224

[20] Sanz ĩ, Lee FEH. 2010. B cells as therapeutic targets in SLE. Nat Rev Rheumatol. 6:326–337. 20520647 10.1038/nrrheum.2010.68 PMC3934759

[21] Mathian A, Gallegos M, Pascual V, Banchereau J, Koutouzov S. 2011. Interferon-α induces unabated production of short-lived plasma cells in pre-autoimmune lupus-prone (NZB×NZW) F1 mice but not in BALB/c mice. Eur J Immunol. 41:863–872. 21312191 10.1002/eji.201040649 PMC3073415

[22] Soni C, et al 2020. Plasmacytoid dendritic cells and type I interferon promote extrafollicular B cell responses to extracellular self-DNA. Immunity. 52:1022–1038. 32454024 10.1016/j.immuni.2020.04.015 PMC7306002

[23] Chen TT, et al 2016. STAT1 regulates marginal zone B cell differentiation in response to inflammation and infection with blood-borne bacteria. J Exp Med. 213:3025–3039. 27849553 10.1084/jem.20151620 PMC5154933

[24] Gaudette BT, et al 2021. Resting innate-like B cells leverage sustained notch2/mTORC1 signaling to achieve rapid and mitosis-independent plasma cell differentiation. J Clin Invest. 131:e151975. 34473651 10.1172/JCI151975 PMC8516456

[25] Giordani L, et al 2009. IFN-α amplifies human naïve B cell TLR-9-mediated activation and Ig production. J Leukoc Biol. 86:261–271. 19401392 10.1189/jlb.0908560

[26] Thibault DL, et al 2009. Type I interferon receptor controls B-cell expression of nucleic acid-sensing Toll-like receptors and autoantibody production in a murine model of lupus. Arthritis Res Ther. 11:R112. 19624844 10.1186/ar2771 PMC2745794

[27] Goodnow CC, Vinuesa CG, Randall KL, MacKay F, Brink R. 2010. Control systems and decision making for antibody production. Nat Immunol. 11:681–688. 20644574 10.1038/ni.1900

[28] Shi W, et al 2015. Transcriptional profiling of mouse B cell terminal differentiation defines a signature for antibody-secreting plasma cells. Nat Immunol. 16:663–673. 25894659 10.1038/ni.3154

[29] Shaffer AL, et al 2002. Blimp-1 orchestrates plasma cell differentiation by extinguishing the mature B cell gene expression program. Immunity. 17:51–62. 12150891 10.1016/s1074-7613(02)00335-7

[30] Scharer CD, et al 2020. Antibody-secreting cell destiny emerges during the initial stages of B-cell activation. Nat Commun. 11:3989. 32778653 10.1038/s41467-020-17798-x PMC7417592

[31] Duffy KR, et al 2012. Activation-induced B cell fates are selected by intracellular stochastic competition. Science. 335:338–341. 22223740 10.1126/science.1213230

[32] Patterson DG, et al 2021. An IRF4–MYC–mTORC1 integrated pathway controls cell growth and the proliferative capacity of activated B cells during B cell differentiation in vivo. J Immunol. 207:1798–1811. 34470852 10.4049/jimmunol.2100440 PMC8455452

[33] Sciammas R, et al 2006. Graded expression of interferon regulatory factor-4 coordinates isotype switching with plasma cell differentiation. Immunity. 25:225–236. 16919487 10.1016/j.immuni.2006.07.009

[34] Ochiai K, et al 2013. Transcriptional regulation of germinal center B and plasma cell fates by dynamical control of IRF4. Immunity. 38:918–929. 23684984 10.1016/j.immuni.2013.04.009 PMC3690549

[35] Sintes J, et al 2017. mTOR intersects antibody-inducing signals from TACI in marginal zone B cells. Nat Commun. 8:1462. 29133782 10.1038/s41467-017-01602-4 PMC5684130

[36] Jones DD, et al 2016. MTOR has distinct functions in generating versus sustaining humoral immunity. J Clin Invest. 126:4250–4261. 27760048 10.1172/JCI86504 PMC5096901

[37] Nojima H, et al 2003. The mammalian target of rapamycin (mTOR) partner, raptor, binds the mTOR substrates p70 S6 kinase and 4E-BP1 through their TOR signaling (TOS) motif. J Biol Chem. 278:15461–15464. 12604610 10.1074/jbc.C200665200

[38] Benhamron S, Pattanayak SP, Berger M, Tirosh B. 2015. mTOR activation promotes plasma cell differentiation and bypasses XBP-1 for immunoglobulin secretion. Mol Cell Biol. 35:153–166. 25332234 10.1128/MCB.01187-14 PMC4295374

[39] Weichhart T, Hengstschläger M, Linke M. 2015. Regulation of innate immune cell function by mTOR. Nat Rev Immunol. 15:599–614. 26403194 10.1038/nri3901 PMC6095456

[40] Zeng H, Chi H. 2014. mTOR signaling and transcriptional regulation in T lymphocytes. Transcription. 5:e28263. 25764218 10.4161/trns.28263 PMC4214230

[41] Limon JJ, Fruman DA. 2012. Akt and mTOR in B cell activation and differentiation. Front Immunol. 3:228. 22888331 10.3389/fimmu.2012.00228 PMC3412259

[42] Akkaya M, et al 2017. B cells produce type 1 IFNs in response to the TLR9 agonist CpG-A conjugated to cationic lipids. J Immunol. 199:931–940. 28652397 10.4049/jimmunol.1700348 PMC5531204

[43] Lin WHW, et al 2015. Asymmetric PI3K signaling driving developmental and regenerative cell fate bifurcation. Cell Rep. 13:2203–2218. 26628372 10.1016/j.celrep.2015.10.072 PMC4685001

[44] Miyakoda M, et al 2018. Differential requirements for IRF4 in the clonal expansion and homeostatic proliferation of naive and memory murine CD8+ T cells. Eur J Immunol. 48:1319–1328. 29745988 10.1002/eji.201747120

[45] Yao S, et al 2013. Interferon regulatory factor 4 sustains CD8+ T cell expansion and effector differentiation. Immunity. 39:833–845. 24211184 10.1016/j.immuni.2013.10.007 PMC3855863

[46] Kallies A, et al 2004. Plasma cell ontogeny defined by quantitative changes in Blimp-1 expression. J Exp Med. 200:967–977. 15492122 10.1084/jem.20040973 PMC2211847

[47] Roederer M . 2011. Interpretation of cellular proliferation data: avoid the panglossian. Cytometry Part A. 79A:95–101. 10.1002/cyto.a.21010 21265003

[48] Alpern D, et al 2019. BRB-seq: ultra-affordable high-throughput transcriptomics enabled by bulk RNA barcoding and sequencing. Genome Biol. 20:71. 30999927 10.1186/s13059-019-1671-x PMC6474054

[49] Sato S, et al 2019. Biochemical analysis of nucleosome targeting by Tn5 transposase. Open Biol. 9:190116. 31409230 10.1098/rsob.190116 PMC6731594

[50] Picelli S, et al 2014. Tn5 transposase and tagmentation procedures for massively scaled sequencing projects. Genome Res. 24:2033–2040. 25079858 10.1101/gr.177881.114 PMC4248319

[51] Ge SX, Son EW, Yao R. 2018. iDEP: an integrated web application for differential expression and pathway analysis of RNA-Seq data. BMC Bioinformatics. 19:534. 30567491 10.1186/s12859-018-2486-6 PMC6299935

[52] Zhou Y, et al 2019. Metascape provides a biologist-oriented resource for the analysis of systems-level datasets. Nat Commun. 10:1523. 30944313 10.1038/s41467-019-09234-6 PMC6447622

[53] Mullan KA, et al 2021. ggVolcanor: a shiny app for customizable visualization of differential expression datasets. Comput Struct Biotechnol J. 19:5735–5740. 34745458 10.1016/j.csbj.2021.10.020 PMC8551465

