# Supplementary material for: Type I interferon promotes the fate of Toll-like receptor 9–stimulated follicular B cells to plasma cell differentiation
Source: PNAS Nexus. 2024 Apr 17;3(4):pgae152. doi: 10.1093/pnasnexus/pgae152 (PMC11042664; doi:10.1093/pnasnexus/pgae152)
Supplement: pgae152_Supplementary_Data [file pgae152_supplementary_data.pdf]

Supplementary Figure 1

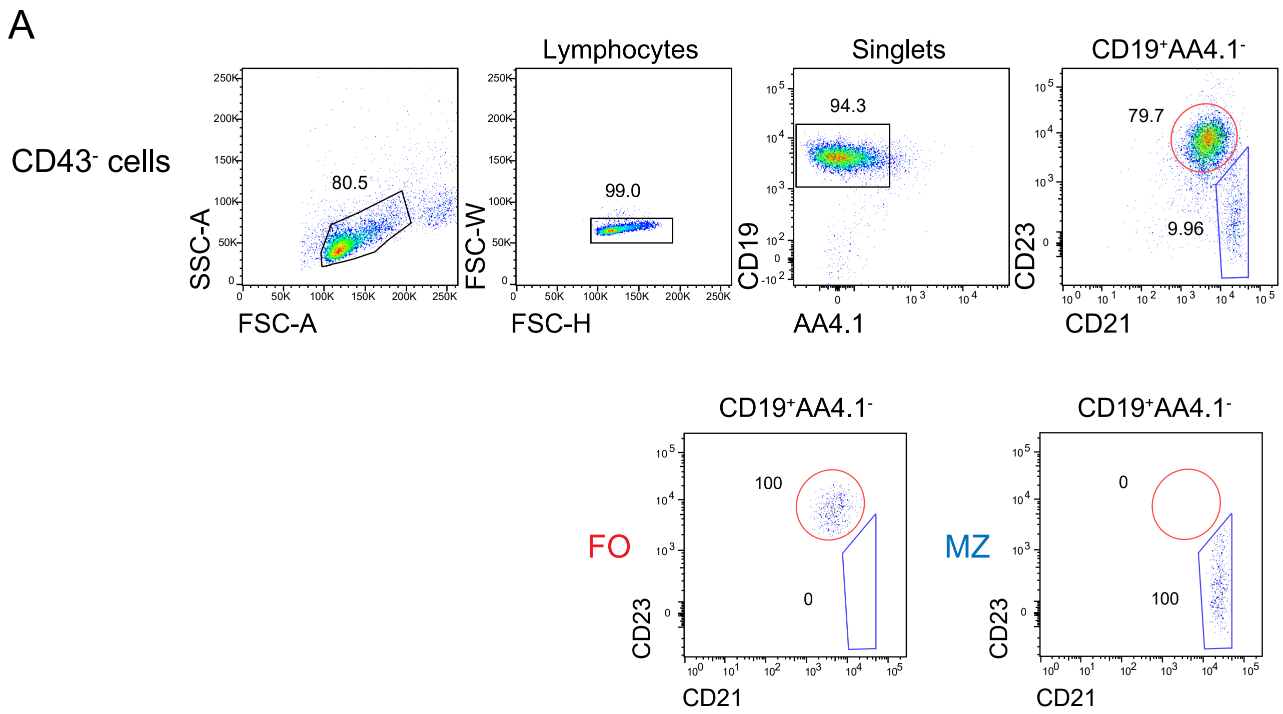

Supplementary Figure 1

(**A**) Gating strategy of FO B cells and MZ B cells sorted by FACS Melody.

Supplementary Figure 2

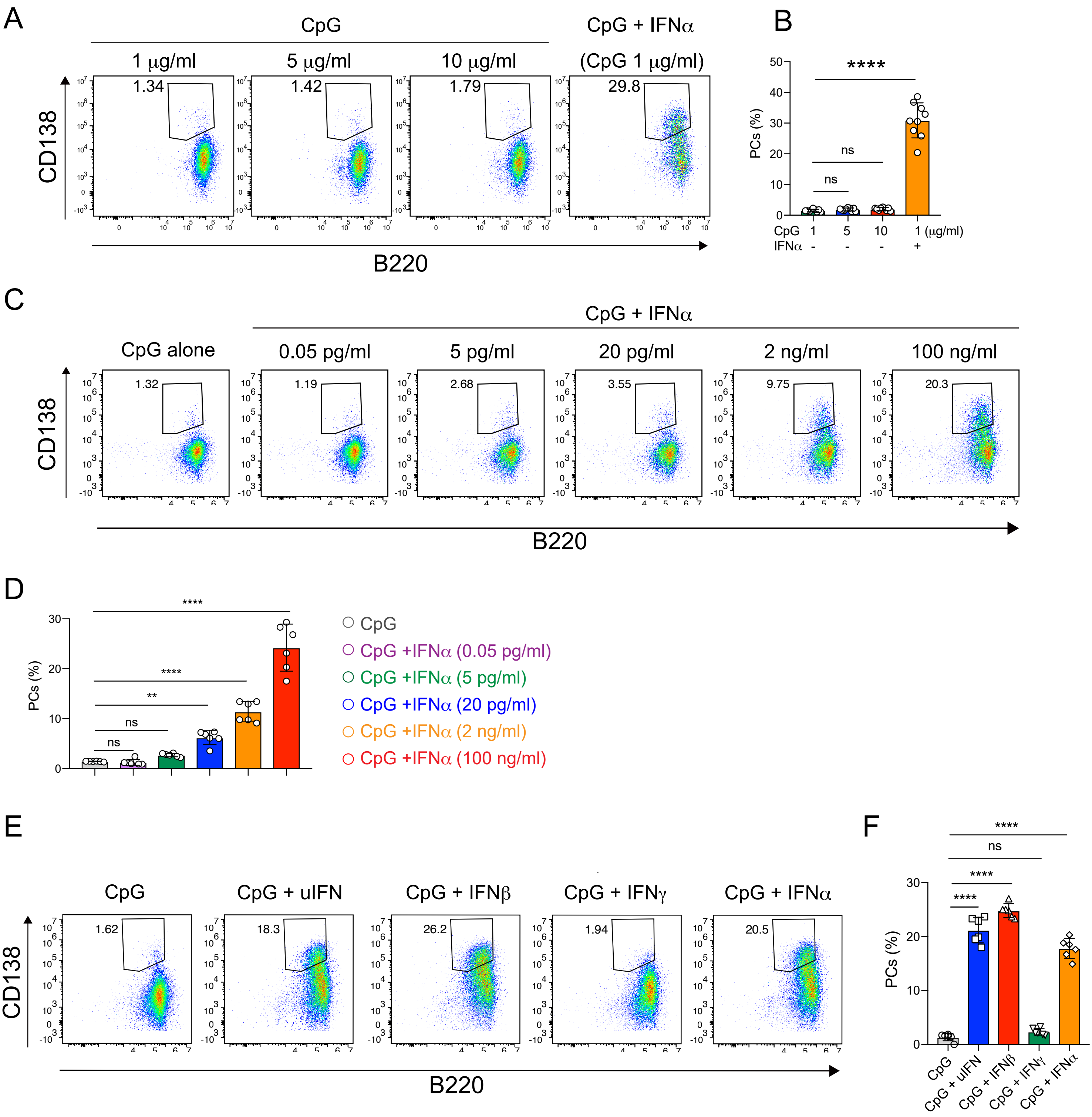

Supplementary Figure 2

(**A**) Representative flow cytometry plots of FO B cells stimulated for 3 days with CpG (1, 5 or 10 μg/ml) or CpG (1 μg/ml) plus IFNα (0.1 μg/ml). Percentages of $CD138^{+}B220^{+/low}$ cells (PCs) are shown. (**B**) Percentages of PCs in (A). (**C**) Representative flow cytometry plots of FO B cells stimulated for 3 days with CpG (1 μg/ml) or CpG (1 μg/ml) plus IFNα (0.05, 5, 20 pg/ml, 2 or 100 ng/ml). Percentages of PCs are shown. (**D**) Percentages of PCs in (C). (**E**) Representative flow cytometry plots of FO B cells stimulated for 3 days with CpG (1 μg/ml) in the presence or absence of IFNα (0.1 μg/ml), universal IFN (0.1 μg/ml), IFNβ (0.1 μg/ml), IFNγ (0.05 μg/ml). Percentages of $CD138^{+}B220^{+/low}$ cells (PCs) are shown. (**F**) Percentages of PCs in (E). Data are pooled from two independent experiments performed in triplicates (B, D and F). Data are presented as mean ± SD. ns, not significant. **P < 0.01, and ****P < 0.001 by one-way ANOVA (B, D and F).

Supplementary Figure 3

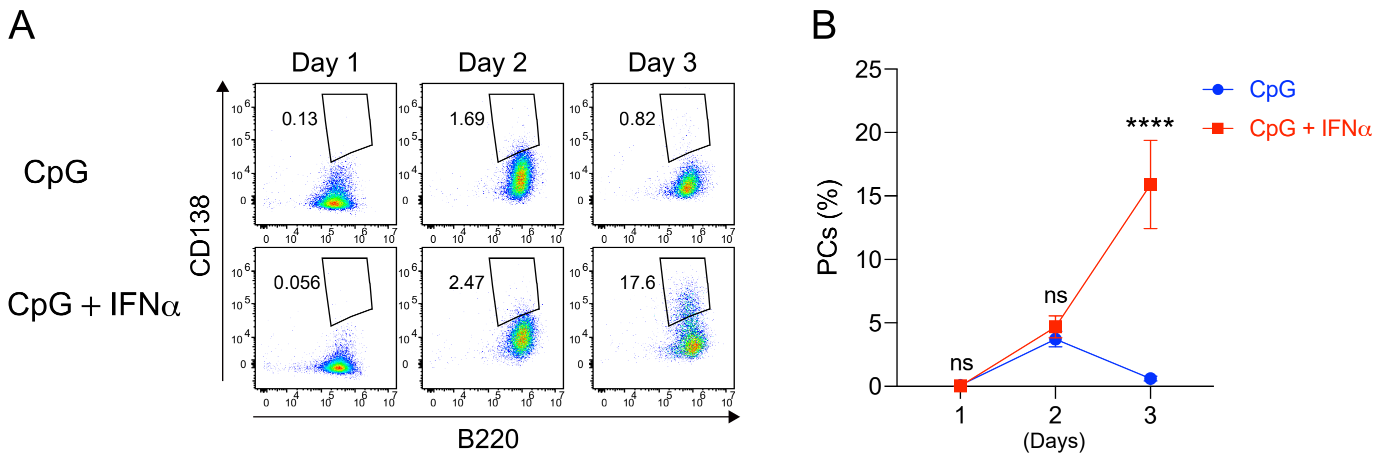

Supplementary Figure 3

(A) Representative flow cytometry plots of FO B cells harvested from the spleen of wild-type mice 1 - 3 days after culture with CpG (1 μg/ml) or CpG (1 μg/ml) plus IFNα (0.1 μg/ml). Percentages of $CD138^{+}B220^{+/low}$ (PCs) are shown. **(B)** Percentages of PCs in (A). Data are pooled from three independent experiments performed in triplicates (B). Data are presented as mean ± SD. ns, not significant. ****P < 0.001 by Student's *t* test (B).

Supplementary Figure 4

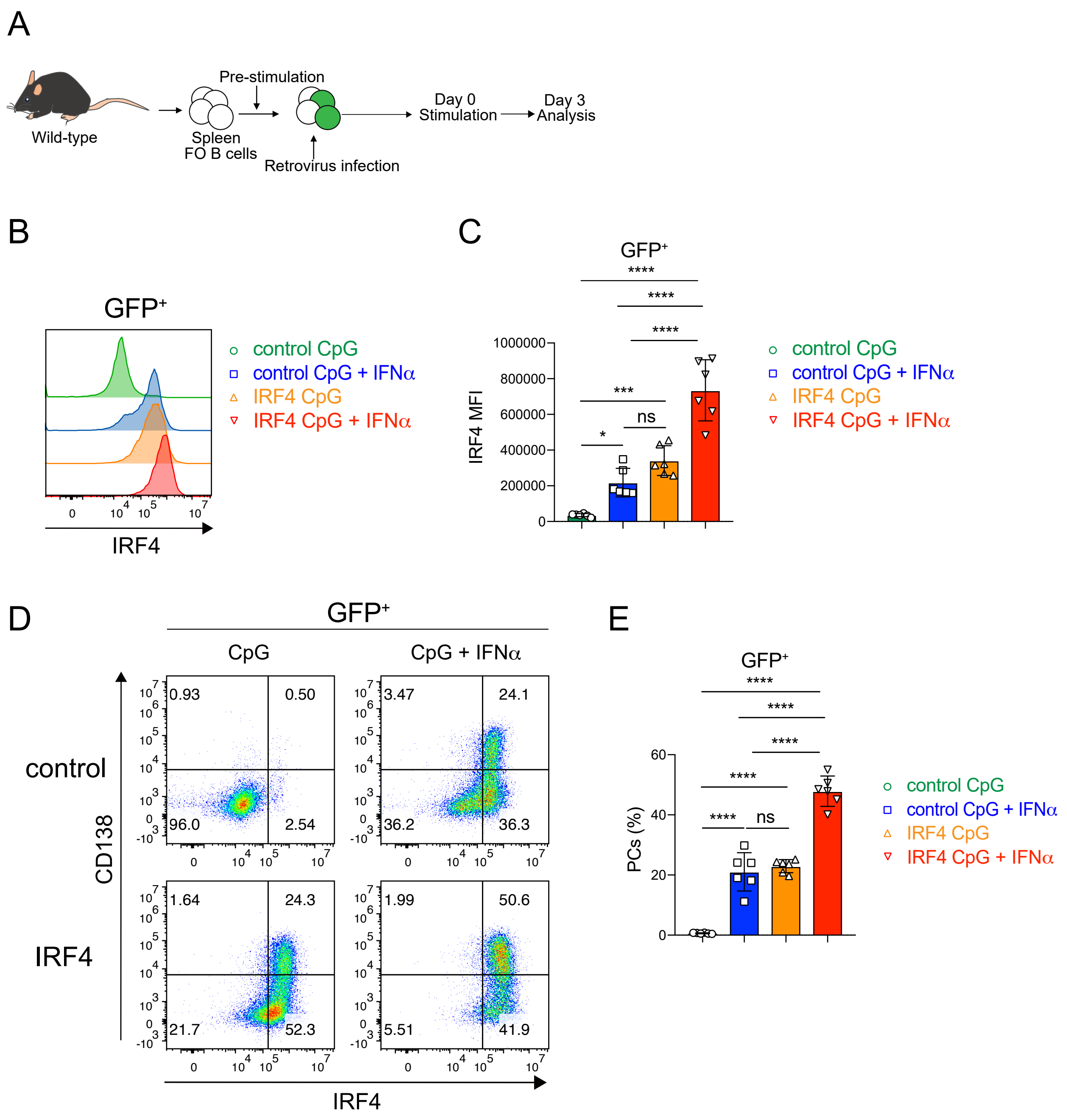

Supplementary Figure 4

(**A**) Schematic of retroviral transduction into FO B cells harvested from the spleen of wild-type mice. (**B**) Representative histogram of IRF4 expression in FO B cells stimulated with CpG (1 µg/ml) or CpG (1 µg/ml) plus IFNα (0.1 µg/ml) after retroviral transduction with GFP (control) or IRF4 vector. Transduced cells were identified by GFP fluorescence. (**C**) MFI of IRF4 expression of FO B cells in (B). (**D**) Flow cytometry analysis of IRF4^{+}CD138^{+} cell (PC) differentiation after retroviral transduction. Transduced cells were identified by GFP fluorescence. (**E**) Percentages of PCs in (D). Data are pooled from two independent experiments performed in triplicates (C and E). Data are presented as mean ± SD. ns, not significant. *P < 0.05, ***P < 0.005, and ****P < 0.001 by one-way ANOVA (C and E).

Supplementary Figure 5

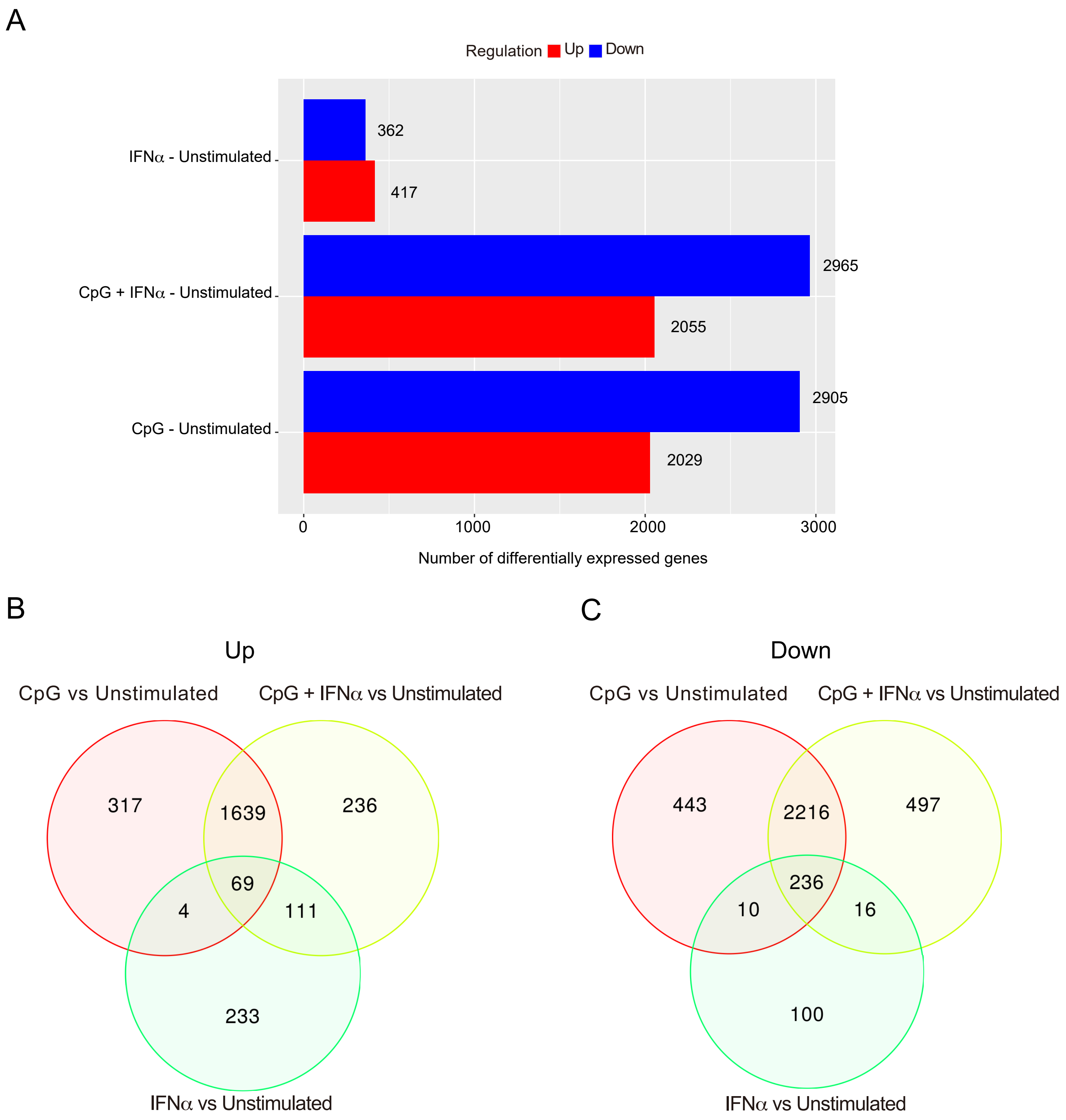

Supplementary Figure 5

(**A**) The number of differentially expressed genes (DEGs) of FO B cells stimulated with IFNα (0.1 μg/ml), CpG (1 μg/ml) or CpG (0.1 μg/ml) plus IFN(α0.1μ g/ml) for 12 hours compared to unstimulated FO B cells (Unstimulated) are indicated. These data were analyzed under the conditions of FDR cutoff < 0.1, and fold change > 2. (**B and C**) Venn diagram of DEGs of up regulated (B) or down regulated (C) genes in (A).

Supplementary Figure 6

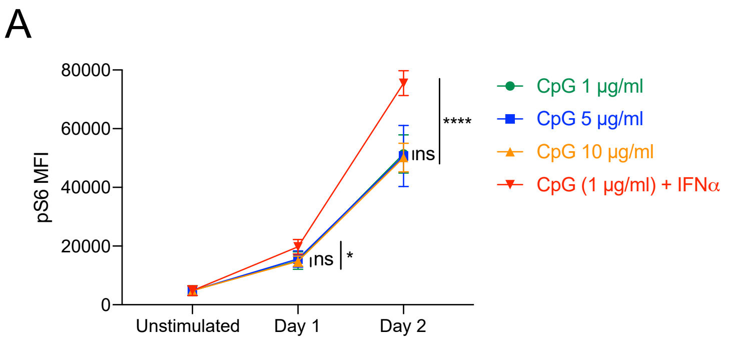

Supplementary Figure 6

(**A**) Mean Fluorescence Intensity (MFI) of pS6 in the representative histogram of pS6 in FO B cells unstimulated (Unstimulated) or stimulated with CpG (1, 5 or 10 μg/ml) or CpG (0.1 μg/ml) plus IFNα (0.1 μg/ml). Data are pooled from two independent experiments performed in triplicates (A). Data are presented as mean ± SD. ns, not significant. **P < 0.01, ***P < 0.005 by one-way ANOVA (A).

Supplementary Figure 7

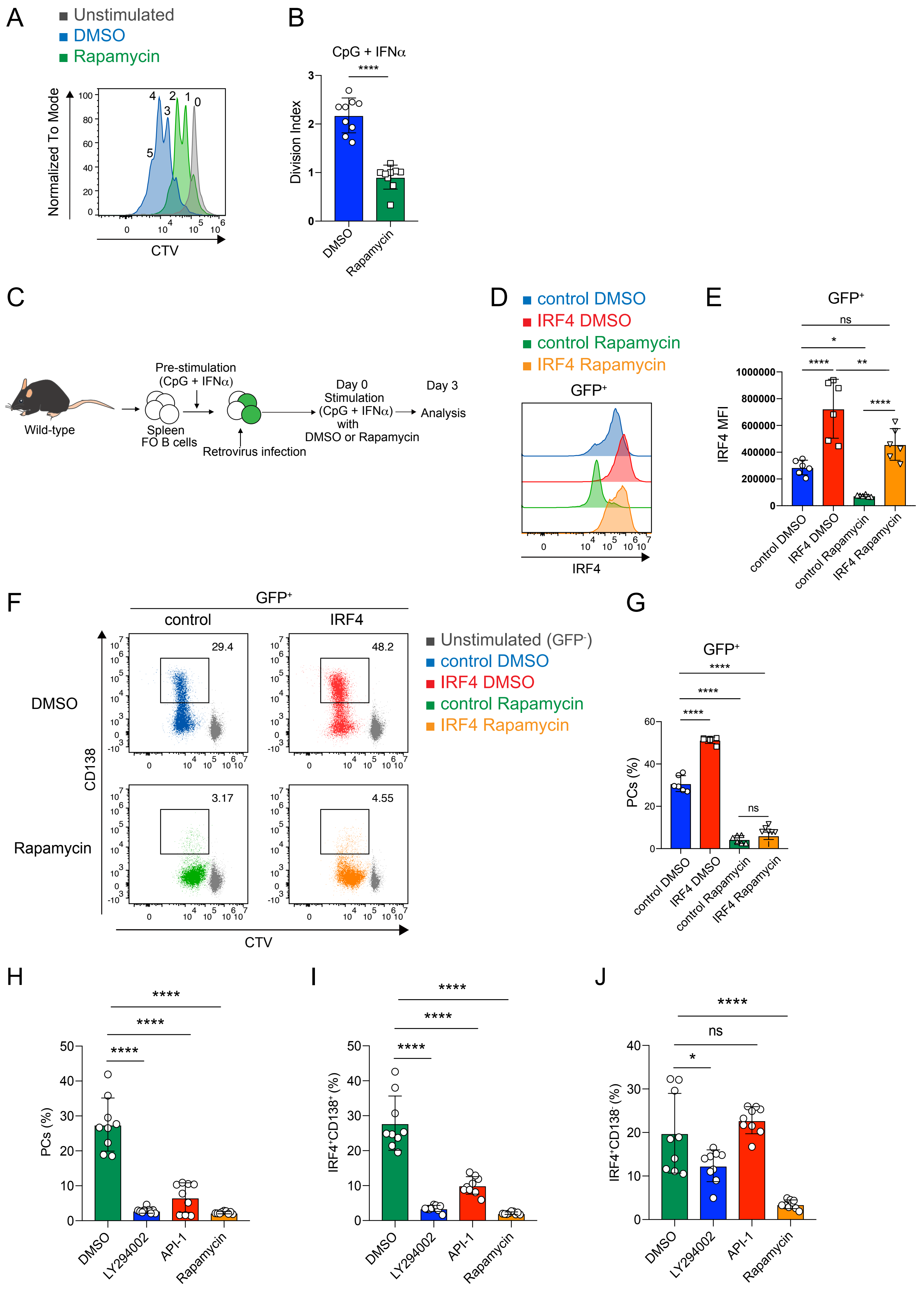

Supplementary Figure 7

(**A**) Representative histogram of Cell Trace Violet (CTV) – stained FO B cells stimulated for 3 days with CpG (1 μg/ml) plus IFNα (0.1 μg/ml) in the presence of DMSO or Rapamycin. (**B**) Division Index in (A). (**C**) The scheme of the experiment of FO B cells harvested from the spleen of wild-type mice transduced with GFP (control) or IRF4 is shown. (**D**) Representative histogram of IRF4 expression in FO B cells after retroviral transduction. Transduced cells were identified by GFP fluorescence. (**E**) MFI of IRF4 expression of FO B cells in (D). (**F**) Flow cytometry analysis of PC differentiation after retroviral transduction. Transduced cells were identified by GFP fluorescence. (**G**) Percentages of PCs in (F). (**H to J**) Percentages of $CD138^{+}B220^{+/low}$ (PCs) (H) and $IRF4^{+}CD138^{+}$ (**I**) and $IRF4^{+}CD138^{-}$ (J) of FO B cells stimulated with CpG (1 μg/ml) plus IFNα (0.1 μg/ml) in the presence or absence of the following inhibitors: (5 μM) LY294002 (PI3K inhibitor), (500 nM) API-1 (Akt inhibitor) or (5 nM) Rapamycin (mTORC1 inhibitor) from spleen of wild-type mice 3 days after culture. Data are pooled from three (B, H to J) or two (E, G) independent experiments performed in triplicates. Data are presented as mean ± SD. ns, not significant. *P < 0.05, **P < 0.01, ****P < 0.001 by Student's *t* test (B) or one-way ANOVA (E, G, H to J).

Supplementary Table 1.

Regulation	Ensembl ID	log2 Fold Change	Adj.Pval	Symbol	Chr	Type	CpG_01	CpG_02	CpG_03	CpGIFN_01	CpGIFN_02	CpGIFN_03
Up	ENSMUSG00000034855	7.375774359	1.55E-45	Cxcl10	5q	protein_coding	2.999484686	2.805714048	2.582784511	8.900069082	9.123477676	8.86836972
Up	ENSMUSG00000073490	7.119681916	3.66E-07	Ifi207	1q	protein_coding	2.321721991	2	2	5.682954614	5.751878421	5.802602984
Up	ENSMUSG00000038507	6.992269703	7.47E-07	Parp12	6q	protein_coding	2	2	2	4.964149438	4.456773008	4.853569902
Up	ENSMUSG00000074896	6.470315144	1.34E-20	Ifit3	19q	protein_coding	2.999484686	2	2.320621695	7.055471171	7.23524085	7.412854324
Up	ENSMUSG00000043953	6.288463384	2.59E-05	Ccrl2	9q	protein_coding	2	2	2	4.096205315	4.167397795	4.317835052
Up	ENSMUSG00000023341	6.223427033	1.55E-07	Mx2	16q	polymorphic_pseudogene	2	2.321162586	2.320621695	5.46981884	5.951156135	5.776614433
Up	ENSMUSG00000035692	6.179941387	7.25E-88	Isg15	4q	protein_coding	3.906134738	3.697788147	4.082461386	9.751149227	9.454149313	9.705712786
Up	ENSMUSG00000033355	6.178601729	2.14E-36	Rtp4	16q	protein_coding	2.999484686	2.998085466	2.582784511	7.887745129	8.008027621	7.907856312
Up	ENSMUSG00000111118	6.137357383	7.13E-58	Gm6545	19q	processed_pseudogene	2.806913235	2.805714048	3.90209467	8.503148525	8.662117894	8.773005725
Up	ENSMUSG00000052776	5.96670226	4.56E-05	Oas1a	5q	protein_coding	2.321721991	2	2	4.710110229	4.456773008	4.949739613
Up	ENSMUSG00000094796	5.945056785	5.07E-05	BC147527	13q	protein_coding	2	2.321162586	2	4.710110229	4.456773008	4.902455941
Up	ENSMUSG00000028037	5.922648967	5.18E-05	Ifi44	3q	protein_coding	2	2	2.320621695	4.468783477	4.904074284	4.639558189
Up	ENSMUSG00000025492	5.887633607	5.56E-66	Ifitm3	7q	protein_coding	4.391483012	4.389216426	4.387022303	10.15985833	9.821723011	9.928593504
Up	ENSMUSG00000032661	5.87410177	1.40E-34	Oas3	5q	protein_coding	2.999484686	2.805714048	2.99673178	7.805632708	7.633439942	7.865334241
Up	ENSMUSG00000000386	5.852847543	5.32E-25	Mx1	16q	polymorphic_pseudogene	2.999484686	2.321162586	2.804554045	7.055471171	7.618867805	7.164949296
Up	ENSMUSG00000014599	5.799582834	2.42E-06	Csf1	3q	protein_coding	2	2.321162586	2.320621695	5.46981884	5.423317318	5.165376458
Up	ENSMUSG00000027514	5.653040904	4.79E-73	Zbp1	2q	protein_coding	4.086674639	3.582409223	3.166293189	8.77950179	8.797679002	8.756480366
Up	ENSMUSG00000025743	5.604609793	5.74E-06	Sdc3	4q	protein_coding	2.321721991	2.321162586	2	5.180082207	5.041538379	5.317322604
Up	ENSMUSG00000020641	5.265625611	2.88E-56	Rsad2	12q	protein_coding	3.699726157	3.456994497	3.45527078	8.135380038	8.220795123	8.298727527
Up	ENSMUSG00000039236	5.262332394	3.92E-122	Isg20	7q	protein_coding	4.522710468	4.389216426	4.638361344	9.614989694	9.384786631	9.534068256
Up	ENSMUSG00000104713	5.222652718	9.12E-04	Gbp6	5q	protein_coding	2.321721991	2	2	4.096205315	4.319328618	3.903139083
Up	ENSMUSG00000040328	5.221911436	1.67E-03	Olfr56	11q	protein_coding	2	2	2.320621695	3.815522598	4.58225461	3.803700975
Up	ENSMUSG00000030107	5.17674572	6.93E-74	Usp18	6q	protein_coding	4.642990356	3.582409223	3.90209467	8.982874742	8.744973038	9.022827164
Up	ENSMUSG00000066677	5.122722575	2.98E-17	Ifi208	1q	protein_coding	3.321309696	2	2.320621695	6.654822891	6.244848674	6.29892221
Up	ENSMUSG00000033880	5.110668581	9.95E-51	Lgals3bp	11q	protein_coding	2.806913235	4.318863619	3.318005342	8.140542325	8.052081284	8.348091421
Up	ENSMUSG00000034459	5.086386953	1.64E-30	Ifit1	19q	protein_coding	4.458588281	3.582409223	3.90209467	8.782807173	8.492641	8.862204534
Up	ENSMUSG00000048534	5.070458936	1.21E-03	Jaml	9q	protein_coding	2	2.321162586	2	3.915275444	3.997563127	4.083550619
Up	ENSMUSG00000039304	4.901714694	3.48E-03	Tnfsf10	3q	protein_coding	2	2	2.320621695	4.17881646	3.698190443	3.696898338
Up	ENSMUSG00000073491	4.884039392	9.52E-46	Ifi213	1q	protein_coding	3.806618702	3.456994497	2.99673178	7.77276335	7.69725221	7.674462018
Up	ENSMUSG00000062488	4.772794585	1.13E-08	Ifit3b	19q	protein_coding	2.321721991	2	2.804554045	5.180082207	5.354619329	5.454779533
Up	ENSMUSG00000048852	4.770991143	9.18E-03	Gm12185	11q	protein_coding	2	2	2	3.592587101	2.805962927	2.997443209
Up	ENSMUSG00000025498	4.733575445	1.29E-83	Irf7	7q	protein_coding	4.754009449	4.0845337	4.316695384	8.795953494	9.02468085	8.736396783
Up	ENSMUSG00000020638	4.705208035	2.80E-12	Cmpk2	12q	protein_coding	2.584618978	2.321162586	2.804554045	5.791953919	5.751878421	5.877872494
Up	ENSMUSG00000044703	4.636662781	2.82E-48	Phf11a	14q	protein_coding	3.906134738	3.582409223	4.082461386	8.268660962	7.95099871	7.966507139
Up	ENSMUSG00000027078	4.601947562	5.78E-03	Ube2l6	2q	protein_coding	2	2	2.320621695	4.008574848	3.457364234	3.456176734
Up	ENSMUSG00000078616	4.600901443	5.16E-03	Trim30c	7q	protein_coding	2	2.321162586	2	3.466710519	3.997563127	3.456176734
Up	ENSMUSG00000045932	4.599677396	2.02E-21	Ifit2	19q	protein_coding	2.806913235	3.456994497	2.582784511	6.640318723	6.611592565	6.709322706
Up	ENSMUSG00000114761	4.536055161	3.30E-06	Gm47242	19q	lncRNA	2	3.167797584	2.99673178	6.239633645	6.105462988	6.243079221
Up	ENSMUSG00000079017	4.532368545	6.90E-111	Ifi27l2a	12q	protein_coding	4.953298547	5.084082535	4.901220968	9.163626705	9.320950772	9.480739791
Up	ENSMUSG00000029392	4.528711514	5.95E-07	Rilpl1	5q	protein_coding	2.806913235	2	2.320621695	5.295656743	4.855179638	5.165376458
Up	ENSMUSG00000070501	4.523549495	3.14E-24	Ifi214	1q	protein_coding	2.806913235	2.805714048	3.45527078	6.669182691	6.817044055	6.580057998
Up	ENSMUSG00000037921	4.46367022	3.22E-07	Ddx60	8q	protein_coding	2.321721991	2.583686427	2.320621695	4.710110229	5.389377191	4.995522638
Up	ENSMUSG00000029561	4.433086931	7.81E-23	Oasl2	5q	protein_coding	3.321309696	2.998085466	2.804554045	6.314626438	6.751757927	6.709322706
Up	ENSMUSG00000073489	4.426433185	2.67E-11	Ifi204	1q	protein_coding	2.584618978	2.321162586	2.99673178	5.817963911	5.48889221	5.925955388
Up	ENSMUSG00000082292	4.417371688	5.47E-16	Gm12250	11q	processed_pseudogene	2.999484686	2.321162586	3.166293189	6.140054613	6.244848674	6.29892221
Up	ENSMUSG00000040483	4.374764942	4.88E-48	Xaf1	11q	protein_coding	4.169123322	3.456994497	4.082461386	7.906048387	7.991154396	7.789390125
Up	ENSMUSG00000078763	4.344497645	5.79E-07	Slfn1	11q	protein_coding	2.806913235	2	2.320621695	4.867832777	5.126404746	4.853569902
Up	ENSMUSG00000014773	4.323324764	3.06E-02	Dll1	17q	protein_coding	2	2	2	3.328792046	2.321278665	2.997443209
Up	ENSMUSG00000078922	4.322129847	2.20E-02	Tgtp1	11q	protein_coding	2	2	2	3.005722225	2.998375876	2.805163665
Up	ENSMUSG00000026896	4.266308598	1.03E-14	Ifih1	2q	protein_coding	2.999484686	2.805714048	2.99673178	6.119286627	6.551477223	5.902114248
Up	ENSMUSG00000046999	4.244297366	1.92E-19	1110032F04Rik	3q	protein_coding	3.169352419	2.805714048	3.802683767	6.818370395	6.951051187	6.853039664
Up	ENSMUSG00000078921	4.214599705	3.03E-03	Tgtp2	11q	protein_coding	2.321721991	2	2.320621695	4.17881646	3.997563127	3.903139083
Up	ENSMUSG00000017830	4.109849302	2.68E-56	Dhx58	11q	protein_coding	4.458588281	4.244903402	4.082461386	7.786001093	8.201365183	8.174861688
Up	ENSMUSG00000054404	4.107158132	6.74E-32	Slfn5	11q	protein_coding	3.906134738	3.319630349	3.318005342	7.200934676	6.962638322	7.082495513
Up	ENSMUSG00000012519	4.091169388	9.56E-04	Mlkl	8q	protein_coding	2.321721991	2.321162586	2.320621695	4.008574848	4.58225461	4.519335248
Up	ENSMUSG00000054072	4.031784276	5.51E-02	Iigp1	18q	protein_coding	2	2	2	2.588779838	2.805962927	2.997443209
Up	ENSMUSG00000070327	4.016600407	1.47E-74	Rnf213	11q	protein_coding	4.458588281	5.041027487	4.518158293	8.305897657	8.640638648	8.498764114
Up	ENSMUSG00000046805	4.013436688	1.33E-36	Mpeg1	19q	protein_coding	3.806618702	4.0845337	3.802683767	7.647817767	7.480638406	7.361328384
Up	ENSMUSG00000078349	3.973962738	1.25E-03	AW011738	4q	lncRNA	2.321721991	2.321162586	2.320621695	4.17881646	4.084978146	4.580698662
Up	ENSMUSG00000068245	3.911988709	3.73E-05	Phf11d	14q	protein_coding	2.584618978	2.321162586	2.320621695	4.710110229	4.697690128	4.317835052
Up	ENSMUSG00000006310	3.910969387	1.99E-14	Zbtb32	7q	protein_coding	3.458775732	2.805714048	3.45527078	6.258750455	6.389222276	6.736540524
Up	ENSMUSG00000041827	3.904409036	3.19E-115	Oasl1	5q	protein_coding	5.356621791	5.520063931	5.386398068	9.235347416	9.213517653	9.137025235
Up	ENSMUSG00000026536	3.895161758	6.27E-16	Ifi211	1q	protein_coding	3.321309696	2.998085466	3.45527078	6.625667255	6.282318989	6.454546534
Up	ENSMUSG00000037849	3.880628755	6.93E-48	Ifi206	1q	protein_coding	4.69956754	4.640638319	4.082461386	8.22072146	8.030222597	8.103480641
Up	ENSMUSG00000022906	3.750867067	7.43E-39	Parp9	16q	protein_coding	3.584275374	4.0845337	4.242763777	7.37746823	7.439768214	7.280415559
Up	ENSMUSG00000002227	3.749203436	5.82E-16	Mov10	3q	protein_coding	2.584618978	3.456994497	3.318005342	6.239633645	5.804337234	6.35268397
Up	ENSMUSG00000078853	3.737032777	7.94E-25	Igtp	11q	protein_coding	3.584275374	3.456994497	3.695912517	6.711423927	6.655090259	6.972324562
Up	ENSMUSG00000091144	3.73263845	6.83E-09	Phf11c	14q	protein_coding	2.806913235	2.583686427	2.804554045	5.258151306	5.354619329	5.28080987
Up	ENSMUSG00000029798	3.725894834	3.55E-23	Herc6	6q	protein_coding	3.321309696	3.456994497	3.580603227	6.437157349	6.536048671	6.776428025
Up	ENSMUSG00000040296	3.722343786	9.80E-36	Ddx58	4q	protein_coding	4.321103504	3.804619095	3.318005342	7.150650414	7.166765301	7.204473811
Up	ENSMUSG00000086782	3.70817063	8.82E-02	E130102H24Rik	4q	lncRNA	2	2	2.320621695	3.17628163	2.805962927	3.318859402
Up	ENSMUSG00000034422	3.645486837	1.53E-41	Parp14	16q	protein_coding	4.391483012	4.166945738	3.695912517	7.314914543	7.406217414	7.510693783
Up	ENSMUSG00000057596	3.604576477	4.50E-69	Trim30d	7q	protein_coding	5.458494546	5.125889818	5.123488531	8.573543873	8.823327075	8.698834634
Up	ENSMUSG00000046879	3.60055098	1.81E-37	Irgm1	11q	protein_coding	4.857092407	4.581770197	4.801747719	8.259199654	8.041193339	8.113896723
Up	ENSMUSG00000032344	3.56488065	6.78E-05	Cgas	9q	protein_coding	2.321721991	2.321162586	2.804554045	4.256952261	4.58225461	4.802969074
Up	ENSMUSG00000078920	3.545157791	3.91E-58	Ifi47	11q	protein_coding	4.999098079	5.041027487	5.279530892	8.644302408	8.336640093	8.490793716
Up	ENSMUSG00000030921	3.544459772	3.52E-161	Trim30a	7q	protein_coding	6.568868213	6.388668499	6.666138664	10.04617742	10.1013605	9.94618731
Up	ENSMUSG00000075602	3.364758172	7.94E-98	Ly6a	15q	protein_coding	6.227842873	6.66874418	6.260583354	9.640721003	9.662101866	9.79742121
Up	ENSMUSG00000035208	3.362470873	2.49E-63	Slfn8	11q	protein_coding	5.321000396	4.950859671	4.801747719	8.135380038	8.414640491	8.13961259
Up	ENSMUSG00000040033	3.347684299	3.68E-23	Stat2	10q	protein_coding	3.906134738	3.582409223	3.580603227	6.470336243	6.566742522	6.681581508
Up	ENSMUSG00000022014	3.340591979	1.48E-25	Epsti1	14q	protein_coding	3.906134738	3.804619095	3.90209467	6.953566325	6.829754097	6.609800982
Up	ENSMUSG00000029298	3.332946862	5.14E-20	Gbp9	5q	protein_coding	3.584275374	3.456994497	3.580603227	6.29624052	6.536048671	6.165091697
Up	ENSMUSG00000069892	3.325606368	6.90E-08	9930111J21Rik2	11q	protein_coding	2.806913235	3.167797584	3.166293189	5.009999048	6.166855675	5.243348998
Up	ENSMUSG00000038037	3.291962674	1.37E-13	Socs1	16q	protein_coding	3.699726157	3.319630349	3.318005342	6.239633645	5.854955295	6.387443434
Up	ENSMUSG00000079227	3.287316112	8.08E-07	Ccr5	9q	protein_coding	3.321309696	3.456994497	2.582784511	5.682954614	5.72491592	5.750149125
Up	ENSMUSG00000086213	3.278123337	1.19E-07	A330040F15Rik	19q	lncRNA	2.999484686	2.583686427	2.804554045	5.219644765	4.804569588	5.082947796
Up	ENSMUSG00000046718	3.274412092	3.54E-94	Bst2	8q	protein_coding	7.237401242	7.135828578	7.707826581	10.61501894	10.52521284	10.72960354
Up	ENSMUSG00000044583	3.182053696	1.80E-14	Tlr7	Xq	protein_coding	3.458775732	3.582409223	3.318005342	5.964883982	6.105462988	6.144917827
Up	ENSMUSG00000067203	3.09248802	7.80E-02	H2-K2	17q	transcribed_unprocessed_pse	2	2.321162586	2.320621695	3.466710519	2.998375876	3.318859402
Up	ENSMUSG00000025647	3.08775023	5.90E-148	Shisa5	9q	protein_coding	7.391311162	7.225089386	7.048935817	10.30370719	10.19888479	10.33919356
Up	ENSMUSG00000024079	3.075171814	9.58E-49	Eif2ak2	17q	protein_coding	5.247005242	5.041027487	5.164109705	8.071947326	8.120917881	8.039353681
Up	ENSMUSG00000035517	3.042915642	1.53E-09	Tdrd7	4q	protein_coding	3.458775732	2.805714048	2.99673178	5.436627907	5.319003353	5.454779533
Up	ENSMUSG00000026104	2.9469808	4.09E-70	Stat1	1q	protein_coding	6.265809295	6.040795015	6.059936843	8.942065925	9.057470225	8.994921935
Up	ENSMUSG00000109408	2.941491354	2.61E-06	A930037H05Rik	11q	lncRNA	2.999484686	3.697788147	2.582784511	4.710110229	5.48889221	5.802602984
Up	ENSMUSG00000034171	2.91156307	1.53E-02	Faah	4q	protein_coding	2.584618978	2.583686427	2	3.815522598	3.90450792	3.696898338
Up	ENSMUSG00000091649	2.862751188	1.13E-53	Phf11b	14q	protein_coding	6.583974653	6.281767347	6.436701155	9.317411639	9.149057636	9.259357827
Up	ENSMUSG00000032690	2.851939763	2.21E-03	Oas2	5q	protein_coding	2.584618978	2.805714048	2	3.328792046	4.167397795	4.317835052
Up	ENSMUSG00000036986	2.841259024	2.71E-57	Pml	9q	protein_coding	5.356621791	5.520063931	5.453483883	8.150811814	8.110539072	8.238123547
Up	ENSMUSG00000041481	2.811637246	9.96E-03	Serpina3g	12q	protein_coding	2.806913235	2	2.320621695	3.328792046	3.805034184	3.996163113
Up	ENSMUSG00000018899	2.770287225	1.56E-08	Irf1	11q	protein_coding	3.699726157	2.583686427	2.99673178	5.258151306	5.389377191	5.082947796
Up	ENSMUSG00000022584	2.769856013	4.86E-06	Ly6c2	15q	protein_coding	3.699726157	2.805714048	2.804554045	5.258151306	4.58225461	5.695715944
Up	ENSMUSG00000026222	2.720282573	2.98E-71	Sp100	1q	protein_coding	5.753933071	5.854413958	5.948075532	8.49107463	8.516421636	8.45437176
Up	ENSMUSG00000027555	2.716057731	1.77E-06	Car13	3q	protein_coding	3.321309696	3.167797584	3.695912517	5.46981884	5.423317318	5.802602984
Up	ENSMUSG00000022901	2.681002192	6.13E-11	Cd86 ENSMUSG0	16q	protein_coding	3.806618702	3.582409223	3.166293189	5.791953919	5.879613546	5.487190656
Up	ENSMUSG00000060519	2.656923271	6.84E-54	Tor3a	1q	protein_coding	5.905928527	5.97369189	6.163745469	8.718672102	8.431415764	8.631558029
Up	ENSMUSG00000032596	2.648107677	3.91E-11	Uba7	9q	protein_coding	3.584275374	3.697788147	3.45527078	5.46981884	5.951156135	5.802602984
Up	ENSMUSG00000043263	2.645970206	7.99E-53	Ifi209	1q	protein_coding	5.929774142	5.581450578	5.748829392	8.333207721	8.235197756	8.369983083
Up	ENSMUSG00000029322	2.613771503	5.12E-54	Plac8	5q	protein_coding	6.374058313	5.903314741	6.222624587	8.640663541	8.75166755	8.779563229

Up	ENSMUSG00000022946	2.605899925	1.94E-06	Dop1b	16q	protein_coding	2.999484686	3.582409223	2.804554045	5.219644765	4.804569588	5.243348998
Up	ENSMUSG00000010358	2.598483677	1.10E-69	Ifi35	11q	protein_coding	6.780366398	6.353907536	6.501539047	9.090822507	9.092170737	9.124201519
Up	ENSMUSG00000042726	2.586695061	1.36E-30	Trafd1	5q	protein_coding	4.905997267	5.166519626	4.801747719	7.350990911	7.336680261	7.478812226
Up	ENSMUSG00000057143	2.544151969	1.40E-42	Trim12c	7q	protein_coding	5.391384814	5.422790001	5.316039959	7.718563221	7.764993946	7.913829861
Up	ENSMUSG00000079363	2.521284869	7.85E-17	Gbp4	5q	protein_coding	4.642990356	3.997127245	4.1648379	6.403197414	6.778227948	6.43806109
Up	ENSMUSG00000009035	2.489093206	2.64E-15	Tmem184b	15q	protein_coding	4.391483012	3.997127245	4.454079762	6.502769217	6.488747621	6.564953354
Up	ENSMUSG00000056290	2.470917768	1.40E-16	Ms4a4b	19q	protein_coding	4.458588281	4.804071306	4.242763777	6.711423927	6.791283034	6.802419904
Up	ENSMUSG00000069874	2.429311863	6.93E-06	Irgm2	11q	protein_coding	3.169352419	3.697788147	3.166293189	5.295656743	4.804569588	5.549907023
Up	ENSMUSG00000001444	2.396206031	1.99E-05	Tbx21	11q	protein_coding	3.584275374	3.582409223	3.580603227	5.180082207	5.751878421	5.580271584
Up	GM4070	2.384109481	6.25E-02	NA	NANA	NA	2.321721991	2.321162586	2.804554045	3.815522598	3.997563127	2.997443209
Up	ENSMUSG00000047735	2.381466159	1.67E-31	Samd9l	6q	protein_coding	4.953298547	4.854678486	5.517588308	7.550335972	7.423090342	7.14477342
Up	ENSMUSG00000023206	2.366032869	3.48E-02	Il15ra	2q	protein_coding	2.321721991	2.583686427	2.99673178	3.328792046	4.319328618	4.165945811
Up	ENSMUSG00000074151	2.365122161	2.11E-25	Nlrc5	8q	protein_coding	4.806471413	5.041027487	4.994275748	7.011066275	7.282235567	7.164949296
Up	ENSMUSG00000027834	2.363000612	2.74E-03	Serpini1	3q	protein_coding	2.584618978	2.998085466	2.582784511	4.468783477	4.084978146	3.803700975
Up	ENSMUSG00000004952	2.339370722	1.07E-05	Rasa4	5q	protein_coding	3.584275374	3.456994497	3.318005342	4.867832777	5.640866387	5.28080987
Up	ENSMUSG00000066258	2.326393048	1.12E-24	Trim12a	7q	protein_coding	5.16900876	4.950859671	5.123488531	7.109121719	7.480638406	7.184846903
Up	ENSMUSG00000071068	2.319721737	1.70E-16	Treml2	17q	protein_coding	4.458588281	3.697788147	4.242763777	6.119286627	6.263705477	6.223973995
Up	ENSMUSG00000062007	2.319074822	5.25E-12	Hsh2d	8q	protein_coding	4.169123322	4.318863619	3.90209467	5.941412971	6.126218854	6.404514076
Up	ENSMUSG00000056116	2.246003386	3.73E-26	H2-T22	17q	protein_coding	5.208534028	5.354094656	4.901220968	7.140379773	7.244763057	7.437943237
Up	ENSMUSG00000002325	2.243909871	8.79E-34	Irf9	14q	protein_coding	5.490913941	5.668894616	5.801279467	7.71164276	8.024705782	7.667407751
Up	ENSMUSG00000049502	2.237224533	5.11E-24	Dtx3l	16q	protein_coding	5.458494546	5.244499708	5.164109705	7.573423469	7.186663621	7.44621018
Up	ENSMUSG00000079339	2.226257391	5.13E-03	Ifit1bl1	19q	protein_coding	3.458775732	2.998085466	2.320621695	4.331072792	4.456773008	4.696110183
Up	ENSMUSG00000063268	2.210597844	2.20E-11	Parp10	15q	protein_coding	3.906134738	4.166945738	4.518158293	6.076831664	6.063033352	6.334985055
Up	ENSMUSG00000072825	2.206918004	1.45E-02	Cep170b	12q	protein_coding	2.999484686	2.583686427	2.99673178	4.401570636	4.456773008	3.903139083
Up	ENSMUSG00000040253	2.180113875	2.38E-07	Gbp7	3q	protein_coding	3.906134738	3.456994497	3.695912517	5.258151306	5.456477304	5.695715944
Up	ENSMUSG00000045868	2.130598119	2.95E-03	Gvin1	7q	protein_coding	2.806913235	2.998085466	2.99673178	4.401570636	4.520877634	3.996163113
Up	ENSMUSG00000096727	2.108555387	7.93E-32	Psmb9	17q	protein_coding	5.699488225	6.062486617	6.059936843	7.881592075	7.968345326	8.098244263
Up	ENSMUSG00000030966	2.106299784	4.42E-06	Trim21	7q	protein_coding	2.806913235	3.904081742	3.802683767	5.180082207	5.126404746	5.454779533
Up	ENSMUSG00000019866	2.100292036	1.67E-03	Crybg1	10q	protein_coding	2.806913235	2.805714048	3.166293189	4.468783477	4.389686971	3.996163113
Up	ENSMUSG00000032265	2.088077368	1.11E-06	Tent5a	9q	protein_coding	3.584275374	2.998085466	3.695912517	4.91679474	5.206555	5.082947796
Up	ENSMUSG00000029605	2.074849674	8.69E-03	Oas1b	5q	polymorphic_pseudogene	2.999484686	3.167797584	2.320621695	4.096205315	3.997563127	4.388175583
Up	ENSMUSG00000037321	2.065194869	1.47E-48	Tap1	17q	protein_coding	6.85698574	7.051566208	6.801045284	8.808981102	9.120894557	8.85601289
Up	ENSMUSG00000071350	2.063410884	2.41E-08	Setdb2	14q	protein_coding	4.321103504	3.997127245	4.1648379	5.817963911	6.336760594	5.639148187
Up	ENSMUSG00000026946	2.056224156	1.90E-24	Nmi	2q	protein_coding	5.671475404	5.520063931	5.694400288	7.478770268	7.633439942	7.667407751
Up	ENSMUSG00000029156	2.048448261	1.28E-05	Sgcb	5q	protein_coding	4.169123322	3.456994497	3.90209467	5.295656743	5.927700603	5.454779533
Up	ENSMUSG00000106734	2.040094484	1.65E-05	Gm20559	6q	lncRNA	2.999484686	3.319630349	3.802683767	4.710110229	4.752119379	5.352934013
Up	GM8995	2.024012541	9.13E-30	NA	NANA	NA	6.168951475	5.950612206	6.623077735	8.071947326	8.476566641	8.144701211
Up	ENSMUSG00000021338	2.005001385	3.92E-03	Carmil1	13q	protein_coding	2.584618978	3.582409223	2.804554045	4.468783477	4.520877634	4.243888402
Up	ENSMUSG00000001123	1.977501983	3.27E-48	Lgals9	11q	protein_coding	7.482808007	7.757790341	7.721500339	9.64617565	9.583606633	9.615127764
Up	ENSMUSG00000029366	1.954253216	1.55E-10	Dck	5q	protein_coding	4.69956754	4.318863619	4.579511348	6.368418777	6.01931795	6.470845728
Up	ENSMUSG00000072620	1.921602784	1.15E-30	Slfn2	11q	protein_coding	7.032423675	7.083744068	6.947864007	8.950908372	8.758331141	9.011729759
Up	ENSMUSG00000039910	1.917145588	2.61E-22	Cited2	10q	protein_coding	5.780403894	6.281767347	5.924623494	7.924122341	7.676293507	7.966507139
Up	ENSMUSG00000015340	1.91711733	1.74E-72	Cybb	Xq	protein_coding	7.767172433	7.703600947	7.768362989	9.740988984	9.55327326	9.631532264
Up	ENSMUSG00000030156	1.901804637	7.88E-36	Cd69	6q	protein_coding	6.553601918	6.504131664	6.260583354	8.381971759	8.249458025	8.257043527
Up	ENSMUSG00000027580	1.885744426	1.85E-33	Helz2	2q	protein_coding	6.458447677	6.300143386	6.241728809	8.066533189	8.166720113	8.266411277
Up	ENSMUSG00000002307	1.881399513	3.66E-31	Daxx	17q	protein_coding	6.188850248	6.018772286	6.436701155	8.145686207	7.867170259	8.119076697
Up	ENSMUSG00000067212	1.857460814	1.72E-13	H2-T23	17q	protein_coding	5.699488225	5.55108373	5.485892117	7.098550035	7.354381283	7.557232812
Up	ENSMUSG00000053835	1.847335295	8.57E-08	H2-T24	17q	protein_coding	4.086674639	3.997127245	4.638361344	5.682954614	6.166855675	5.750149125
Up	ENSMUSG00000021458	1.838989953	1.69E-02	Aopep	13q	protein_coding	3.169352419	3.167797584	3.318005342	4.867832777	4.752119379	3.696898338
Up	ENSMUSG00000050628	1.829511933	3.51E-12	Ubald2	11q	protein_coding	5.284477164	4.804071306	4.801747719	6.738912922	6.778227948	6.486962833
Up	ENSMUSG00000049401	1.822058483	1.30E-28	Ogfr	2q	protein_coding	6.425281903	6.281767347	6.260583354	8.109287832	8.211112863	7.949160884
Up	ENSMUSG00000090272	1.819725717	4.70E-45	Mndal	1q	protein_coding	7.043394554	6.737778795	7.37731752	8.927207001	8.774856697	8.889742728
Up	ENSMUSG00000051727	1.809212665	6.80E-13	Kctd14	7q	protein_coding	5.284477164	5.455948755	5.279530892	7.305752709	7.008078064	6.776428025
Up	ENSMUSG00000052749	1.786369786	6.23E-05	Trim30b	7q	protein_coding	3.999226959	2.998085466	3.695912517	4.468783477	5.041538379	5.421623505
Up	ENSMUSG00000060550	1.782105746	1.57E-33	H2-Q7	17q	protein_coding	7.021368728	6.682817905	6.947864007	8.474817295	8.676261764	8.749816855
Up	ENSMUSG00000018930	1.781934605	9.35E-03	Ccl4	11q	protein_coding	6.657221508	6.472074244	6.123113893	8.259199654	8.110539072	8.129381183
Up	ENSMUSG00000024912	1.770359344	2.06E-02	Fosl1	19q	protein_coding	3.458775732	3.697788147	3.695912517	4.964149438	5.206555	4.853569902
Up	ENSMUSG00000039997	1.753816892	7.34E-27	Ifi203	1q	protein_coding	6.168951475	5.668894616	6.102360763	7.732305342	7.618867805	7.702339136
Up	ENSMUSG00000033538	1.748070421	8.51E-12	Casp4	9q	protein_coding	5.128370049	4.903570453	5.038645038	6.470336243	6.765053644	6.681581508
Up	ENSMUSG00000026581	1.73731932	3.30E-06	Sell	1q	protein_coding	4.247113775	3.582409223	3.90209467	5.332211814	4.997156581	5.723189241
Up	ENSMUSG00000027698	1.736313436	1.46E-09	Nceh1	3q	protein_coding	5.043488293	4.903570453	5.038645038	6.470336243	6.765053644	6.549688893
Up	ENSMUSG00000022661	1.720833325	3.81E-06	Cd200	16q	protein_coding	4.458588281	4.244903402	4.316695384	5.765466376	6.105462988	5.639148187
Up	ENSMUSG00000041736	1.719642268	4.85E-08	Tspo	15q	protein_coding	4.321103504	4.318863619	4.638361344	5.817963911	6.084404146	5.949408939
Up	ENSMUSG00000042700	1.718404948	2.32E-04	Sipa1l1	12q	protein_coding	3.169352419	3.697788147	3.90209467	4.817150599	5.206555	4.853569902
Up	ENSMUSG00000073409	1.688796283	3.61E-25	H2-Q6	17q	protein_coding	6.458447677	6.710560058	6.241728809	7.988521171	8.277562539	8.098244263
Up	ENSMUSG00000027951	1.683963586	4.16E-11	Adar	3q	protein_coding	5.086553339	4.751624178	5.123488531	6.550091248	6.581847988	6.51866674
Up	ENSMUSG00000027639	1.680681421	1.58E-26	Samhd1	2q	protein_coding	6.107553421	5.903314741	5.948075532	7.534736457	7.551407999	7.72289907
Up	ENSMUSG00000028977	1.656421179	5.82E-03	Casz1	4q	protein_coding	3.458775732	3.456994497	4.1648379	4.710110229	5.520594783	4.802969074
Up	ENSMUSG00000022867	1.615867942	7.73E-22	Usp25	16q	protein_coding	6.356571492	6.973570106	6.608433663	8.25444563	8.410416017	8.050241413
Up	ENSMUSG00000035929	1.615168846	3.30E-06	H2-Q4	17q	protein_coding	4.169123322	4.751624178	4.454079762	5.565042135	6.225742134	5.853216432
Up	ENSMUSG00000039531	1.60202797	4.99E-11	Zup1	10q	protein_coding	5.881682145	6.186203161	6.241728809	7.603643073	7.829697017	7.518555292
Up	ENSMUSG00000045827	1.596729161	6.43E-05	Serpinb9	13q	protein_coding	4.642990356	4.996647896	4.518158293	6.119286627	6.504687576	5.828131653
Up	ENSMUSG00000034765	1.590192148	8.29E-02	Dusp5	19q	protein_coding	2.584618978	2.998085466	3.580603227	4.17881646	4.319328618	3.996163113
Up	ENSMUSG00000026770	1.584213161	5.89E-06	Il2ra	2q	protein_coding	4.806471413	5.125889818	5.203618383	6.420277303	6.697314778	6.421385089
Up	ENSMUSG00000054520	1.575023391	7.03E-05	Sh3bp2	5q	protein_coding	3.906134738	3.997127245	3.802683767	5.139404073	5.245019893	5.204889431
Up	ENSMUSG00000019082	1.553294759	2.16E-15	Slc25a22	7q	protein_coding	6.657221508	6.104915245	6.761881132	8.055643577	7.860991923	8.20440453
Up	ENSMUSG00000052566	1.549013735	4.08E-03	Hook2	8q	protein_coding	3.584275374	4.244903402	4.082461386	5.180082207	5.354619329	5.317322604
Up	ENSMUSG00000079491	1.53823088	5.46E-04	H2-T10	17q	polymorphic_pseudogene	3.906134738	4.0845337	4.1648379	5.367863493	4.804569588	5.723189241
Up	ENSMUSG00000048895	1.513238161	2.15E-04	Cdk5r1	11q	protein_coding	4.69956754	4.0845337	4.749314978	5.893293381	6.01931795	5.723189241
Up	ENSMUSG00000000275	1.477772287	2.43E-15	Trim25	11q	protein_coding	5.881682145	6.125670628	6.038247703	7.42900821	7.318759357	7.557232812
Up	ENSMUSG00000028793	1.467033415	1.53E-08	Rnf19b	4q	protein_coding	5.953232024	5.879071529	5.242073881	7.220567696	7.282235567	6.815242324
Up	ENSMUSG00000022074	1.452432961	3.12E-02	Tnfrsf10b	14q	protein_coding	4.391483012	4.244903402	3.166293189	5.009999048	5.354619329	5.317322604
Up	ENSMUSG00000026158	1.446472789	5.86E-06	Ogfrl1	1q	protein_coding	4.522710468	4.804071306	4.801747719	5.941412971	6.244848674	5.828131653
Up	ENSMUSG00000017929	1.442003769	1.30E-12	B4galt5	2q	protein_coding	7.117939934	6.439288273	6.864045072	8.454234565	7.99680076	8.242876854
Up	GM1966	1.436389508	4.44E-04	NA	NANA	NA	5.458494546	6.244297818	5.826806342	7.230284915	7.589274392	6.840550257
Up	ENSMUSG00000070031	1.417471372	2.90E-11	Sp140	1q	protein_coding	5.391384814	5.422790001	5.826806342	7.022296287	6.962638327	6.695518784
Up	ENSMUSG00000072621	1.386609317	3.52E-02	Slfn10-ps	11q	transcribed_unprocessed_pse	3.906134738	4.854678486	4.852341504	5.565042135	6.01931795	5.877872494
Up	ENSMUSG00000041571	1.362554379	5.88E-18	Selenow	7q	protein_coding	6.583974653	6.535492208	6.38608585	7.766098649	7.816986471	7.895834496
Up	ENSMUSG00000029780	1.361337709	2.81E-10	Nt5c3	6q	protein_coding	6.188850248	5.996408161	6.183640037	7.462372891	7.354381283	7.486848909
Up	ENSMUSG00000022385	1.356811492	2.26E-02	Gtse1	15q	protein_coding	4.247113775	4.244903402	4.387022303	5.332211814	5.55161566	5.487190656
Up	ENSMUSG00000023067	1.354726688	3.63E-07	Cdkn1a	17q	protein_coding	6.806360937	7.625602514	7.122926537	8.268660962	8.741614096	8.616985751
Up	ENSMUSG00000059456	1.329678851	2.95E-13	Ptk2b	14q	protein_coding	6.128311128	6.263154224	5.775292773	7.333065491	7.448035402	7.25240376
Up	ENSMUSG00000026088	1.327154473	1.77E-08	Mitd1	1q	protein_coding	5.831931563	5.803797334	5.548603073	7.191017057	6.791283034	6.972324562
Up	ENSMUSG00000035547	1.326675775	9.26E-02	Capn5	7q	protein_coding	3.169352419	3.456994497	3.318005342	4.17881646	4.456773008	4.083550619
Up	ENSMUSG00000082088	1.326546027	1.31E-05	Gm15753	5q	unprocessed_pseudogene	4.69956754	4.854678486	4.852341504	5.893293381	6.01931795	6.082646289
Up	ENSMUSG00000055413	1.325851705	6.40E-02	H2-Q5	17q	polymorphic_pseudogene	3.999226959	4.244903402	3.695912517	4.817150599	5.167036404	5.243348998
Up	ENSMUSG00000070034	1.31573108	2.95E-10	Sp110	1q	protein_coding	5.553645796	5.803797334	6.059936843	7.011066275	7.115784623	7.093063654
Up	ENSMUSG00000038179	1.315536755	9.91E-12	Slamf7	1q	protein_coding	6.506809153	6.66874418	6.608433663	7.918122813	8.035718396	7.646035601
Up	ENSMUSG00000064215	1.307279534	5.14E-06	Ifi27	12q	protein_coding	5.522620808	5.640331481	5.608701291	6.893679188	6.724793153	6.853039664
Up	ENSMUSG00000061731	1.282898556	1.32E-02	Ext1	15q	protein_coding	4.169123322	4.389216426	4.316695384	5.295656743	5.423317318	5.454779533
Up	ENSMUSG00000026637	1.276952021	5.68E-08	Traf5	1q	protein_coding	4.999098079	5.422790001	5.242073881	6.160527876	6.456329429	6.595006137
Up	ENSMUSG00000025746	1.267898866	7.76E-09	Il6	5q	protein_coding	8.032407932	7.318192397	7.768362989	9.213468403	8.800910058	8.916765102
Up	ENSMUSG00000024308	1.267567924	8.60E-26	Tapbp	17q	protein_coding	7.767172433	7.610956529	7.800928178	9.008512266	9.038433769	8.889742728
Up	ENSMUSG00000000184	1.267436713	1.01E-19	Ccnd2	6q	protein_coding	9.690717694	9.673906767	9.769919087	10.99652977	11.09149338	10.82620038
Up	ENSMUSG00000038059	1.263522203	8.44E-02	Smim3	18q	protein_coding	3.699726157	4.996647896	4.518158293	5.436627907	6.041341223	5.204889431
Up	ENSMUSG00000038400	1.261674121	6.74E-05	Pmepa1	2q	protein_coding	5.208534028	5.751340062	5.279530892	6.565526496	6.842353141	6.404514076
Up	ENSMUSG00000033781	1.259622986	9.73E-04	Asb13	13q	protein_coding	4.458588281	4.640638319	4.454079762	5.738483419	5.520594783	5.61001021
Up	ENSMUSG00000006418	1.229155472	7.35E-12	Rnf114	2q	protein_coding	6.964786469	6.353907536	6.623077735	7.881592075	7.69725221	8.000586227
Up	ENSMUSG00000061232	1.223563316	3.38E-37	H2-K1	17q	protein_coding	10.43776401	10.61822994	10.55857554	11.73421591	11.76576497	11.78401372

Up	ENSMUSG00000024737	1.214798334	1.14E-04	Slc15a3	19q	protein_coding	5.490913941	5.520063931	5.279530892	6.534489072	6.751757927	6.404514076
Up	ENSMUSG00000100394	1.189287558	9.97E-02	Gm28791	1q	lncRNA	3.906134738	4.0845337	3.695912517	4.91679474	4.752119379	4.902455941
Up	ENSMUSG00000049659	1.187282241	1.82E-20	Aftph	11q	protein_coding	7.450203869	7.371300266	7.477418474	8.584949584	8.679776171	8.537968682
Up	ENSMUSG00000026031	1.184446603	5.47E-16	Cflar	1q	protein_coding	7.293616208	7.543144913	7.297402923	8.507150806	8.700684705	8.421206251
Up	ENSMUSG00000073643	1.182603882	2.15E-03	Wdfy1	1q	protein_coding	4.754009449	4.751624178	4.901220968	5.367863493	6.084404146	6.039586705
Up	ENSMUSG00000030530	1.182222029	1.82E-06	Furin	7q	protein_coding	5.553645796	5.581450578	5.548603073	6.725233895	6.626237999	6.667508274
Up	ENSMUSG00000041645	1.179551591	3.17E-21	Ddx24	12q	protein_coding	8.338831921	8.190977619	8.419797157	9.550519487	9.562821129	9.343641488
Up	ENSMUSG00000079455	1.174601176	1.66E-03	Gm16026	1q	transcribed_unprocessed_pse	3.906134738	4.854678486	4.801747719	5.436627907	5.778346251	5.61001021
Up	ENSMUSG00000028466	1.169348256	8.57E-02	Creb3	4q	protein_coding	4.169123322	3.697788147	3.318005342	4.867832777	4.456773008	4.696110183
Up	ENSMUSG00000090523	1.14957323	2.78E-05	Gypc	18q	protein_coding	6.227842873	6.318288305	6.297568938	7.239937121	7.480638406	7.437943237
Up	ENSMUSG00000002728	1.124971992	2.29E-05	Naa20	2q	protein_coding	5.953232024	5.829327619	5.748829392	6.905857143	6.85484311	6.983728044
Up	ENSMUSG00000066036	1.119754107	2.79E-10	Ubr4	4q	protein_coding	6.553601918	6.878941489	6.563587879	7.697701445	7.823355742	7.763160278
Up	ENSMUSG00000042901	1.11766763	4.04E-07	Aida	1q	protein_coding	5.458494546	5.455948755	5.548603073	6.58079835	6.520453337	6.502901874
Up	ENSMUSG00000102882	1.115360523	7.21E-02	Gm2065	9q	TEC	4.905997267	4.45629742	4.852341504	5.765466376	5.389377191	6.017565178
Up	ENSMUSG00000063445	1.114426242	7.85E-03	Nmral1	16q	protein_coding	3.999226959	4.751624178	4.69490454	5.436627907	5.282485818	5.723189241
Up	ENSMUSG00000021242	1.110941569	1.29E-08	Npc2	12q	protein_coding	6.265809295	6.405739863	6.315712134	7.518966423	7.431453378	7.242944313
Up	ENSMUSG00000039501	1.107132131	6.89E-05	Znfx1	2q	protein_coding	5.671475404	5.318480102	5.42033088	6.403197414	6.520453337	6.609800982
Up	ENSMUSG00000034484	1.10580849	6.80E-13	Snx2	18q	protein_coding	7.514691565	6.950488457	7.593504208	8.41642906	8.504580316	8.478754943
Up	D1ERTD622E	1.098334845	1.66E-07	NA	NANA	NA	6.021400456	5.854413958	6.222624587	6.92990894	7.126125899	7.204473811
Up	ENSMUSG00000027293	1.076617671	1.14E-05	Ehd4	2q	protein_coding	5.976314581	5.879071529	5.826806342	6.999748162	6.996851597	6.749959265
Up	ENSMUSG00000060802	1.068393054	2.86E-34	B2m	2q	protein_coding	11.60166938	11.59376038	11.63732053	12.6763065	12.63178984	12.72577954
Up	ENSMUSG00000019876	1.060908208	2.72E-06	Pkib	10q	protein_coding	5.831931563	6.018772286	6.059936843	7.011066275	7.03027227	6.913914601
Up	ENSMUSG00000025036	1.06041099	1.47E-03	Sfxn2	19q	protein_coding	4.857092407	5.125889818	5.203618383	5.987979252	5.951156135	6.165091697
Up	ENSMUSG00000056501	1.054978006	2.54E-02	Cebpb	2q	protein_coding	5.458494546	4.903570453	4.518158293	6.140054613	5.951156135	5.802602984
Up	ENSMUSG00000073411	1.04936337	1.44E-31	H2-D1	17q	protein_coding	10.02964027	10.09026011	9.975049372	11.0868799	11.00518486	11.1414596
Up	ENSMUSG00000034575	1.03765701	6.22E-04	Tent4a	13q	protein_coding	5.490913941	5.354094656	5.123488531	6.385912893	6.336760594	6.144917827
Up	ENSMUSG00000043279	1.035603816	3.44E-07	Trim56	5q	protein_coding	5.953232024	6.225191687	6.36881162	7.077171389	7.380532145	7.082495513
Up	ENSMUSG00000028270	1.022289658	5.97E-02	Gbp2	3q	protein_coding	3.906134738	4.166945738	4.316695384	4.91679474	5.126404746	4.902455941
Up	ENSMUSG00000021886	1.021542464	4.85E-03	Gpr65	12q	protein_coding	5.128370049	4.804071306	5.123488531	5.964883982	6.084404146	5.802602984
Up	ENSMUSG00000038213	1.020802308	1.60E-03	Tapbpl	6q	protein_coding	5.16900876	5.041027487	4.901220968	5.738483419	6.186752756	5.949408939
Up	ENSMUSG00000078771	1.015055919	2.32E-03	Evi2a	11q	protein_coding	4.806471413	4.804071306	4.638361344	5.595435969	5.48889221	5.853216432
Up	ENSMUSG00000020108	1.004939631	4.91E-02	Ddit4	10q	protein_coding	4.391483012	3.997127245	4.316695384	5.139404073	5.282485818	4.802969074
Up	ENSMUSG00000066440	1.004018384	3.69E-02	Zfyve26	12q	protein_coding	4.522710468	4.804071306	4.638361344	5.180082207	5.640866387	5.750149125
Down	ENSMUSG00000025433	-4.022981239	4.38E-02	Crisp3	17q	protein_coding	2.999484686	2.805714048	2.582784511	2	2	2
Down	ENSMUSG00000022468	-3.45398394	6.47E-02	Endou	15q	protein_coding	2.806913235	4.0845337	3.318005342	2.324219709	2.321278665	2
Down	ENSMUSG00000026509	-3.387581391	5.16E-02	Capn2	1q	protein_coding	2.999484686	3.697788147	3.580603227	2	2.583879954	2
Down	ENSMUSG00000042514	-2.949069571	2.37E-04	Klhl14	18q	protein_coding	4.458588281	4.804071306	4.579511348	2.812261074	3.168120306	2
Down	ENSMUSG00000025212	-2.869953008	3.68E-02	Sfxn3	19q	protein_coding	4.086674639	2.998085466	3.166293189	2.324219709	2.321278665	2.320905922
Down	ENSMUSG00000043008	-2.332967803	7.99E-18	Klhl6	16q	protein_coding	6.583974653	6.566185532	6.623077735	4.256952261	4.456773008	4.696110183
Down	ENSMUSG00000044162	-2.250718731	1.40E-03	Tnip3	6q	protein_coding	5.247005242	4.950859671	5.578965098	3.17628163	3.319978926	3.903139083
Down	ENSMUSG00000004698	-2.071911898	3.55E-07	Hdac9	12q	protein_coding	5.753933071	5.996408161	6.059936843	4.256952261	4.084978146	4.083550619
Down	ENSMUSG00000025203	-2.012373605	6.22E-05	Scd2	19q	protein_coding	4.754009449	4.950859671	5.279530892	3.17628163	3.582796595	3.581552438
Down	ENSMUSG00000002944	-1.953780302	1.06E-23	Cd36	5q	protein_coding	7.923798665	7.94459775	7.906454331	5.987979252	5.974236416	6.144917827
Down	ENSMUSG00000106019	-1.949979409	8.26E-02	Gm43672	3q	lncRNA	3.584275374	4.0845337	3.802683767	2.324219709	3.168120306	2.583258477
Down	ENSMUSG00000036880	-1.90150747	5.71E-02	Acaa2	18q	protein_coding	3.458775732	3.997127245	3.90209467	3.005722225	2.805962927	2.320905922
Down	ENSMUSG00000037235	-1.888319728	6.02E-02	Mxd4	5q	protein_coding	3.806618702	3.167797584	3.90209467	2.588779838	2.583879954	2.805163665
Down	ENSMUSG00000020573	-1.869448828	1.21E-05	Pik3cg	12q	protein_coding	4.905997267	5.125889818	5.517588308	3.708356769	3.805034184	3.581552438
Down	ENSMUSG00000024063	-1.81386878	1.99E-17	Lbh	17q	protein_coding	7.382698178	7.28166898	7.509299332	5.868618021	5.520594783	5.580271584
Down	ENSMUSG00000036565	-1.802598096	1.31E-03	Ttyh3	5q	protein_coding	4.458588281	5.041027487	4.316695384	3.328792046	3.168120306	3.456176734
Down	ENSMUSG00000039193	-1.792466662	2.55E-03	Nlrc4	17q	protein_coding	4.806471413	4.166945738	4.994275748	2.588779838	3.457364234	3.803700975
Down	ENSMUSG00000027984	-1.75490404	9.34E-04	Hadh	3q	protein_coding	5.128370049	4.903570453	5.123488531	3.17628163	3.698190443	3.996163113
Down	ENSMUSG00000021990	-1.745373255	2.01E-02	Spata13	14q	protein_coding	3.906134738	3.997127245	4.1648379	2.588779838	2.998375876	3.167083843
Down	ENSMUSG00000051457	-1.71790737	6.35E-02	Spn	7q	protein_coding	3.906134738	3.582409223	4.1648379	2.588779838	2.998375876	2.997443209
Down	ENSMUSG00000020186	-1.714936102	1.23E-02	Csrp2	10q	protein_coding	4.321103504	4.581770197	4.579511348	3.328792046	2.998375876	3.456176734
Down	ENSMUSG00000043168	-1.69540834	1.65E-02	4930426D05Rik	18q	lncRNA	4.086674639	4.854678486	4.1648379	3.328792046	3.319978926	2.997443209
Down	ENSMUSG00000073678	-1.684807569	3.81E-05	Pgap1	1q	protein_coding	5.16900876	5.084082535	5.386398068	3.815522598	3.90450792	3.803700975
Down	ENSMUSG00000038034	-1.680386859	5.04E-02	Igsf8	1q	protein_coding	4.321103504	4.45629742	3.90209467	2.812261074	3.319978926	3.167083843
Down	ENSMUSG00000049858	-1.676862734	2.96E-02	Suox	10q	protein_coding	4.642990356	4.581770197	5.316039959	3.466710519	3.805034184	3.456176734
Down	ENSMUSG00000074272	-1.665186084	4.61E-02	Ceacam1	7q	protein_coding	3.458775732	4.0845337	4.242763777	2.588779838	2.805962927	3.318859402
Down	ENSMUSG00000028634	-1.661696846	1.03E-02	Hivep3	4q	protein_coding	4.642990356	4.903570453	4.901220968	3.328792046	3.805034184	3.456176734
Down	ENSMUSG00000095105	-1.627062575	8.44E-02	Edaradd	13q	protein_coding	3.321309696	3.804619095	4.242763777	3.005722225	2.583879954	2.997443209
Down	ENSMUSG00000071637	-1.617149614	5.33E-04	Cebpd	16q	protein_coding	6.408408649	5.92715729	6.721624073	4.710110229	4.456773008	5.387687525
Down	ENSMUSG00000041429	-1.608352276	8.44E-02	Nthl1	17q	protein_coding	4.391483012	4.244903402	4.579511348	3.17628163	3.168120306	3.456176734
Down	ENSMUSG00000013846	-1.603778706	4.34E-03	St3gal1	15q	protein_coding	4.857092407	4.244903402	4.638361344	3.708356769	2.998375876	3.456176734
Down	ENSMUSG00000020788	-1.602008461	1.73E-03	Atp2a3	11q	protein_coding	4.857092407	4.318863619	4.901220968	3.328792046	3.319978926	3.803700975
Down	ENSMUSG00000037972	-1.599964331	3.04E-04	Snn	16q	protein_coding	4.69956754	4.751624178	4.994275748	3.708356769	3.457364234	3.581552438
Down	ENSMUSG00000003418	-1.589575759	1.19E-03	St8sia6	2q	protein_coding	5.831931563	5.166519626	6.059936843	4.468783477	3.997563127	4.519335248
Down	ENSMUSG00000025959	-1.561695207	1.22E-02	Klf7	1q	protein_coding	4.754009449	4.950859671	4.94849849	3.466710519	4.167397795	3.167083843
Down	ENSMUSG00000026347	-1.556274543	7.36E-03	Tmem163	1q	protein_coding	4.642990356	4.389216426	4.638361344	3.005722225	3.698190443	3.456176734
Down	ENSMUSG00000032398	-1.55550124	3.40E-06	Snapc5	9q	protein_coding	6.065119939	5.803797334	5.971152431	4.594486971	4.520877634	4.580698662
Down	ENSMUSG00000025372	-1.542383593	9.41E-03	Baiap2	11q	protein_coding	5.208534028	4.950859671	4.316695384	4.008574848	3.319978926	3.581552438
Down	ENSMUSG00000040451	-1.519139462	2.65E-04	Sgms1	19q	protein_coding	5.043488293	5.166519626	5.123488531	3.328792046	3.997563127	4.165945811
Down	ENSMUSG00000027435	-1.487428501	2.46E-05	Cd93	2q	protein_coding	6.227842873	6.751197983	6.813867251	4.710110229	5.611725352	5.243348998
Down	ENSMUSG00000038366	-1.480563829	3.32E-02	Lasp1	11q	protein_coding	3.906134738	4.318863619	4.242763777	3.17628163	2.998375876	3.318859402
Down	ENSMUSG00000019982	-1.46895311	6.79E-02	Myb	10q	protein_coding	4.458588281	4.950859671	5.08169039	3.592587101	4.084978146	3.318859402
Down	ENSMUSG00000037946	-1.454914665	1.55E-02	Fgd3	13q	protein_coding	4.642990356	4.804071306	3.802683767	3.328792046	3.457364234	3.456176734
Down	ENSMUSG00000022594	-1.433650293	6.38E-12	Lynx1	15q	protein_coding	7.598903291	7.784140388	7.60829983	6.29624052	6.336760594	6.204612365
Down	ENSMUSG00000006517	-1.431833185	1.02E-02	Mvd	8q	protein_coding	5.208534028	4.996647896	5.123488531	3.708356769	3.698190443	4.317835052
Down	ENSMUSG00000039168	-1.423948243	9.26E-02	Dap	15q	protein_coding	4.169123322	3.582409223	4.082461386	3.17628163	2.998375876	2.997443209
Down	ENSMUSG00000064267	-1.412812885	4.96E-09	Hvcn1	5q	protein_coding	6.338870112	6.263154224	6.183640037	4.468783477	5.126404746	5.204889431
Down	ENSMUSG00000039981	-1.410794379	7.86E-03	Zc3h12d	10q	protein_coding	4.458588281	5.084082535	4.316695384	2.812261074	3.997563127	3.696898338
Down	ENSMUSG00000046668	-1.391938642	1.08E-03	Cxxc5	18q	protein_coding	5.043488293	4.854678486	4.852341504	4.096205315	3.698190443	3.581552438
Down	ENSMUSG00000048924	-1.391581528	5.10E-02	Ccdc125	13q	protein_coding	4.169123322	4.581770197	4.316695384	3.005722225	3.168120306	3.803700975
Down	ENSMUSG00000005540	-1.38935855	7.45E-04	Fcer2a	8q	protein_coding	5.458494546	4.903570453	4.901220968	4.256952261	3.805034184	3.803700975
Down	ENSMUSG00000089715	-1.38649043	1.76E-03	Cbx6	15q	protein_coding	5.321000396	5.422790001	5.548603073	3.815522598	4.58225461	4.243888402
Down	ENSMUSG00000038421	-1.381550969	3.30E-11	Fcrla	1q	protein_coding	6.844495105	6.710560058	6.680211353	5.097545622	5.354619329	5.802602984
Down	ENSMUSG00000062901	-1.376058772	1.54E-05	Klhl24	16q	protein_coding	5.208534028	5.751340062	5.517588308	4.17881646	4.167397795	4.580698662
Down	ENSMUSG00000005447	-1.328883308	2.33E-02	Pafah1b3	7q	protein_coding	4.69956754	4.581770197	4.638361344	3.466710519	3.319978926	3.996163113
Down	ENSMUSG00000044678	-1.324090546	4.91E-04	Ly6k	15q	protein_coding	7.054282635	6.581290742	6.351328047	5.682954614	5.389377191	5.243348998
Down	ENSMUSG00000093661	-1.302186923	9.58E-02	Eif4e3	6q	protein_coding	4.321103504	4.389216426	4.1648379	3.17628163	3.457364234	3.456176734
Down	ENSMUSG00000061273	-1.289777486	1.02E-03	Mmgt1	Xq	protein_coding	5.929774142	5.696903206	5.801279467	4.710110229	4.697690128	4.580698662
Down	ENSMUSG00000026866	-1.28794643	8.11E-02	Kynu	2q	protein_coding	4.458588281	4.244903402	4.316695384	3.466710519	3.457364234	3.318859402
Down	ENSMUSG00000040061	-1.284787469	7.46E-02	Plcb2	2q	protein_coding	4.169123322	4.0845337	4.387022303	3.17628163	3.319978926	3.456176734
Down	ENSMUSG00000026360	-1.280802396	7.93E-02	Rgs2	1q	protein_coding	4.458588281	3.804619095	3.90209467	3.005722225	3.457364234	3.167083843
Down	ENSMUSG00000070283	-1.277985821	2.05E-02	Ndufaf3	9q	protein_coding	5.584017617	4.640638319	4.852341504	4.008574848	4.084978146	3.996163113
Down	ENSMUSG00000024975	-1.267375835	9.17E-08	Pdcd4	19q	protein_coding	6.699448566	6.962075441	6.315712134	5.332211814	5.456477304	5.667709299
Down	ENSMUSG00000022186	-1.256359223	6.41E-05	Oxct1	15q	protein_coding	5.881682145	5.950612206	6.279194665	5.009999048	4.904074284	4.802969074
Down	ENSMUSG00000038481	-1.254258099	1.58E-02	Cdk19	10q	protein_coding	5.043488293	4.318863619	4.69490454	3.466710519	3.90450792	3.803700975
Down	ENSMUSG00000020183	-1.247271703	6.52E-03	Cpm	10q	protein_coding	4.953298547	5.084082535	4.801747719	4.256952261	3.805034184	3.696898338
Down	ENSMUSG00000037649	-1.238046664	4.15E-03	H2-DMa	17q	protein_coding	4.806471413	4.854678486	4.69490454	3.815522598	4.167397795	3.318859402
Down	ENSMUSG00000004665	-1.213192073	2.74E-07	Cnn2	10q	protein_coding	6.356571492	6.336207845	6.260583354	5.436627907	4.752119379	5.317322604
Down	ENSMUSG00000021959	-1.197155603	2.06E-02	Lats2	14q	protein_coding	4.642990356	4.640638319	4.94849849	3.328792046	3.90450792	4.083550619
Down	ENSMUSG00000034111	-1.187879962	4.02E-02	Tmed8	12q	protein_coding	4.999098079	4.751624178	5.038645038	3.915275444	4.167397795	3.803700975
Down	ENSMUSG00000052160	-1.184406286	1.03E-02	Pld4	12q	protein_coding	5.208534028	4.996647896	5.351647879	3.592587101	3.805034184	4.853569902
Down	ENSMUSG00000026616	-1.181012064	2.80E-03	Cr2	1q	protein_coding	5.490913941	5.281964061	6.102360763	4.17881646	4.389686971	5.124750744
Down	ENSMUSG00000022048	-1.180954916	1.18E-03	Dpysl2	14q	protein_coding	5.284477164	5.611191398	5.316039959	4.17881646	4.389686971	4.580698662
Down	ENSMUSG00000057337	-1.173274547	4.12E-05	Chst3	10q	protein_coding	5.553645796	6.125670628	6.059936843	4.964149438	5.041538379	4.580698662
Down	ENSMUSG00000037071	-1.169612017	8.71E-08	Scd1	19q	protein_coding	6.85698574	6.581290742	7.0597411	5.595435969	5.520594783	6.039586705
Down	ENSMUSG00000020601	-1.169478352	1.60E-02	Trib2	12q	protein_coding	4.953298547	5.318480102	4.852341504	3.915275444	4.167397795	4.165945811

Down	ENSMUSG00000031133	-1.167987068	3.57E-04	Arhgef6	Xq	protein_coding	5.699488225	5.724378375	5.85188939	4.17881646	5.084595505	4.750528768
Down	ENSMUSG00000018474	-1.163645396	3.80E-04	Chd3	11q	protein_coding	5.881682145	5.696903206	6.36881162	4.468783477	4.904074284	5.352934013
Down	ENSMUSG00000034634	-1.160968	8.29E-30	Ly6d	15q	protein_coding	11.87587087	11.66417164	11.77400337	10.69351091	10.43555115	10.70052655
Down	ENSMUSG00000055148	-1.155265537	8.35E-04	Klf2	8q	protein_coding	5.671475404	5.829327619	5.548603073	4.533003876	4.752119379	4.696110183
Down	ENSMUSG00000024854	-1.154279022	4.15E-06	Pold4	19q	protein_coding	6.628367471	6.455774391	6.38608585	5.295656743	5.48889221	5.454779533
Down	ENSMUSG00000041959	-1.153007628	1.29E-08	S100a10	3q	protein_coding	7.320923061	7.388577157	7.461209903	6.119286627	6.166855675	6.51866674
Down	ENSMUSG00000024349	-1.132749672	7.92E-03	Sting1	18q	protein_coding	5.391384814	5.166519626	5.038645038	4.331072792	3.90450792	4.455245417
Down	ENSMUSG00000006717	-1.12128164	5.33E-02	Acot13	13q	protein_coding	4.999098079	5.281964061	5.203618383	3.915275444	3.997563127	4.639558189
Down	ENSMUSG00000060090	-1.096547354	1.54E-03	Rp2	Xq	protein_coding	5.753933071	6.062486617	6.059936843	5.180082207	4.951366008	4.750528768
Down	ENSMUSG00000031827	-1.096449153	3.41E-04	Cotl1	8q	protein_coding	5.425329863	5.611191398	5.721871508	4.256952261	4.697690128	4.853569902
Down	ENSMUSG00000023990	-1.094731778	1.53E-02	Tfeb	17q	protein_coding	4.905997267	5.084082535	5.242073881	4.096205315	4.084978146	4.317835052
Down	ENSMUSG00000040247	-1.092192152	1.30E-02	Tbc1d10c	19q	protein_coding	4.522710468	4.996647896	5.08169039	3.592587101	3.997563127	4.317835052
Down	ENSMUSG00000022051	-1.088397192	5.04E-02	Bnip3l	14q	protein_coding	4.391483012	4.996647896	4.518158293	3.708356769	3.582796595	4.083550619
Down	ENSMUSG00000040433	-1.065894688	1.60E-02	Zbtb38	9q	protein_coding	4.999098079	5.318480102	4.579511348	3.815522598	4.319328618	4.165945811
Down	ENSMUSG00000034041	-1.063367773	3.43E-02	Lyl1	8q	protein_coding	4.642990356	4.581770197	4.801747719	3.815522598	3.698190443	3.996163113
Down	ENSMUSG00000038811	-1.055388305	5.74E-02	Gngt2	11q	protein_coding	4.905997267	4.804071306	4.749314978	4.256952261	3.805034184	3.803700975
Down	ENSMUSG00000032216	-1.043729347	2.01E-04	Nedd4	9q	protein_coding	6.713253987	7.062372102	7.091679706	5.817963911	5.974236416	6.103703535
Down	ENSMUSG00000031112	-1.036883375	1.77E-02	Stk26	Xq	protein_coding	5.321000396	5.125889818	5.386398068	4.468783477	4.389686971	4.317835052
Down	ENSMUSG00000056737	-1.031337004	1.85E-04	Capg	6q	protein_coding	5.726967465	6.353907536	6.315712134	4.964149438	4.997156581	5.580271584
Down	ENSMUSG00000000594	-1.031092083	7.15E-06	Gm2a	11q	protein_coding	6.086492685	6.455774391	6.637574655	5.332211814	5.423317318	5.580271584
Down	ENSMUSG00000025534	-1.03015807	1.31E-03	Gusb	5q	protein_coding	6.356571492	6.371392708	6.623077735	5.367863493	5.245019893	5.802602984
Down	ENSMUSG00000024646	-1.023686697	2.80E-02	Cyb5a	18q	protein_coding	4.999098079	5.281964061	5.42033088	4.331072792	4.319328618	4.455245417
Down	ENSMUSG00000032009	-1.022324987	1.54E-05	Sesn3	9q	protein_coding	6.941517226	6.581290742	6.888494348	5.682954614	5.697439905	6.103703535
Down	ENSMUSG00000037922	-1.017901479	1.24E-10	Bank1	3q	protein_coding	7.905876969	8.018595225	8.133100521	6.953566325	7.041242635	7.082495513
Down	ENSMUSG00000051748	-1.017403313	3.63E-02	Wfdc21	11q	protein_coding	6.227842873	6.166306523	5.900783918	5.295656743	5.084595505	5.124750744
Down	ENSMUSG00000031155	-1.015722458	3.11E-13	Pim2	Xq	protein_coding	9.38914429	9.293108993	9.432285795	8.37322729	8.225611994	8.486791943
Down	ENSMUSG00000022475	-1.015033488	4.26E-02	Hdac7	15q	protein_coding	4.754009449	4.903570453	4.801747719	4.096205315	3.582796595	4.243888402
Down	ENSMUSG00000063065	-1.005618127	1.63E-03	Mapk3	7q	protein_coding	6.302802146	6.816483156	6.578691895	5.595435969	5.778346251	5.518889611
Down	ENSMUSG00000030707	-1.003164283	1.10E-17	Coro1a	7q	protein_coding	8.587694536	8.45153933	8.524908504	7.534736457	7.50461607	7.564845425

Supplementary Table 1. Differentially expressed genes of CpG plus IFNα vs CpG

Supplementary Table 2.

Regulation	Ensembl ID	log2 Fold Change	Adj.Pval	Symbol	Chr	Type	CpG_01	CpG_02	CpG_03	CpGIFN_01	CpGIFN_02	CpGIFN_03
Up	ENSMUSG00000073490	7.119681916	3.66E-07	Ifi207	1q	protein_coding	2.321722	2	2	5.682954614	5.75187842	5.80260298
Up	ENSMUSG00000038507	6.992269703	7.47E-07	Parp12	6q	protein_coding	2	2	2	4.964149438	4.45677301	4.8535699
Up	ENSMUSG00000035692	6.179941387	7.25E-88	Isg15	4q	protein_coding	3.906135	3.697788	4.082461	9.751149227	9.45414931	9.70571279
Up	ENSMUSG00000052776	5.96670226	4.56E-05	Oas1a	5q	protein_coding	2.321722	2	2	4.710110229	4.45677301	4.94973961
Up	ENSMUSG00000032661	5.87410177	1.40E-34	Oas3	5q	protein_coding	2.999485	2.805714	2.996732	7.805632708	7.63343994	7.86533424
Up	ENSMUSG00000014599	5.799582834	2.42E-06	Csf1	3q	protein_coding	2	2.321163	2.320622	5.46981884	5.42331732	5.16537646
Up	ENSMUSG00000030107	5.17674572	6.93E-74	Usp18	6q	protein_coding	4.64299	3.582409	3.902095	8.982874742	8.74497304	9.02282716
Up	ENSMUSG00000066677	5.122722575	2.98E-17	Ifi208	1q	protein_coding	3.32131	2	2.320622	6.654822891	6.24484867	6.29892221
Up	ENSMUSG00000039304	4.901714694	3.48E-03	Tnfsf10	3q	protein_coding	2	2	2.320622	4.17881646	3.69819044	3.69689834
Up	ENSMUSG00000073491	4.884039392	9.52E-46	Ifi213	1q	protein_coding	3.806619	3.456994	2.996732	7.77276335	7.69725221	7.67446202
Up	ENSMUSG00000020638	4.705208035	2.80E-12	Cmpk2	12q	protein_coding	2.584619	2.321163	2.804554	5.791953919	5.75187842	5.87787249
Up	ENSMUSG00000027078	4.601947562	5.78E-03	Ube2l6	2q	protein_coding	2	2	2.320622	4.008574848	3.45736423	3.45617673
Up	ENSMUSG00000078616	4.600901443	5.16E-03	Trim30c	7q	protein_coding	2	2.321163	2	3.466710519	3.99756313	3.45617673
Up	ENSMUSG00000070501	4.523549495	3.14E-24	Ifi214	1q	protein_coding	2.806913	2.805714	3.455271	6.669182691	6.81704406	6.580058
Up	ENSMUSG00000029561	4.433086931	7.81E-23	Oasl2	5q	protein_coding	3.32131	2.998085	2.804554	6.314626438	6.75175793	6.70932271
Up	ENSMUSG00000073489	4.426433185	2.67E-11	Ifi204	1q	protein_coding	2.584619	2.321163	2.996732	5.817963911	5.48889221	5.92595539
Up	ENSMUSG00000026896	4.266308598	1.03E-14	Ifih1	2q	protein_coding	2.999485	2.805714	2.996732	6.119286627	6.55147722	5.90211425
Up	ENSMUSG00000054072	4.031784276	5.51E-02	Iigp1	18q	protein_coding	2	2	2	2.588779838	2.80596293	2.99744321
Up	ENSMUSG00000070327	4.016600407	1.47E-74	Rnf213	11q	protein_coding	4.458588	5.041027	4.518158	8.305897657	8.64063865	8.49876411
Up	ENSMUSG00000041827	3.904409036	3.19E-115	Oasl1	5q	protein_coding	5.356622	5.520064	5.386398	9.235347416	9.21351765	9.13702524
Up	ENSMUSG00000026536	3.895161758	6.27E-16	Ifi211	1q	protein_coding	3.32131	2.998085	3.455271	6.625667255	6.28231899	6.45454653
Up	ENSMUSG00000037849	3.880628755	6.93E-48	Ifi206	1q	protein_coding	4.699568	4.640638	4.082461	8.22072146	8.0302226	8.10348064
Up	ENSMUSG00000022906	3.750867067	7.43E-39	Parp9	16q	protein_coding	3.584275	4.084534	4.242764	7.37746823	7.43976821	7.28041556
Up	ENSMUSG00000002227	3.749203436	5.82E-16	Mov10	3q	protein_coding	2.584619	3.456994	3.318005	6.239633645	5.80433723	6.35268397
Up	ENSMUSG00000078853	3.737032777	7.94E-25	Igtp	11q	protein_coding	3.584275	3.456994	3.695913	6.711423927	6.65509026	6.97232456
Up	ENSMUSG00000029798	3.725894834	3.55E-23	Herc6	6q	protein_coding	3.32131	3.456994	3.580603	6.437157349	6.53604867	6.77642803
Up	ENSMUSG00000034422	3.645486837	1.53E-41	Parp14	16q	protein_coding	4.391483	4.166946	3.695913	7.314914543	7.40621741	7.51069378
Up	ENSMUSG00000057596	3.604576477	4.50E-69	Trim30d	7q	protein_coding	5.458495	5.12589	5.123489	8.573543873	8.82332708	8.69883463
Up	ENSMUSG00000046879	3.60055098	1.81E-37	Irgm1	11q	protein_coding	4.857092	4.58177	4.801748	8.259199654	8.04119334	8.11389672
Up	ENSMUSG00000030921	3.544459772	3.52E-161	Trim30a	7q	protein_coding	6.568868	6.388668	6.666139	10.04617742	10.1013605	9.94618731
Up	ENSMUSG00000038037	3.291962674	1.37E-13	Socs1	16q	protein_coding	3.699726	3.31963	3.318005	6.239633645	5.8549553	6.38744343
Up	ENSMUSG00000024079	3.075171814	9.58E-49	Eif2ak2	17q	protein_coding	5.247005	5.041027	5.16411	8.071947326	8.12091788	8.03935368
Up	ENSMUSG00000026104	2.9469808	4.09E-70	Stat1	1q	protein_coding	6.265809	6.040795	6.059937	8.942065925	9.05747023	8.99492194
Up	ENSMUSG00000032690	2.851939763	2.21E-03	Oas2	5q	protein_coding	2.584619	2.805714	2	3.328792046	4.1673978	4.31783505
Up	ENSMUSG00000036986	2.841259024	2.71E-57	Pml	9q	protein_coding	5.356622	5.520064	5.453484	8.150811814	8.11053907	8.23812355
Up	ENSMUSG00000041481	2.811637246	9.96E-03	Serpina3g	12q	protein_coding	2.806913	2	2.320622	3.328792046	3.80503418	3.99616311
Up	ENSMUSG00000018899	2.770287225	1.56E-08	Irf1	11q	protein_coding	3.699726	2.583686	2.996732	5.258151306	5.38937719	5.0829478
Up	ENSMUSG00000026222	2.720282573	2.98E-71	Sp100	1q	protein_coding	5.753933	5.854414	5.948076	8.49107463	8.51642164	8.45437176
Up	ENSMUSG00000022901	2.681002192	6.13E-11	Cd86	16q	protein_coding	3.806619	3.582409	3.166293	5.791953919	5.87961355	5.48719066
Up	ENSMUSG00000032596	2.648107677	3.91E-11	Uba7	9q	protein_coding	3.584275	3.697788	3.455271	5.46981884	5.95115614	5.80260298
Up	ENSMUSG00000043263	2.645970206	7.99E-53	Ifi209	1q	protein_coding	5.929774	5.581451	5.748829	8.333207721	8.23519776	8.36998308
Up	ENSMUSG00000042726	2.586695061	1.36E-30	Trafd1	5q	protein_coding	4.905997	5.16652	4.801748	7.350990911	7.33668026	7.47881223
Up	ENSMUSG00000057143	2.544151969	1.40E-42	Trim12c	7q	protein_coding	5.391385	5.42279	5.31604	7.718563221	7.76499395	7.91382986
Up	ENSMUSG00000079363	2.521284869	7.85E-17	Gbp4	5q	protein_coding	4.64299	3.997127	4.164838	6.403197414	6.77822795	6.43806109
Up	ENSMUSG00000069874	2.429311863	6.93E-06	Irgm2	11q	protein_coding	3.169352	3.697788	3.166293	5.295656743	4.80456959	5.54990702
Up	ENSMUSG00000001444	2.396206031	1.99E-05	Tbx21	11q	protein_coding	3.584275	3.582409	3.580603	5.180082207	5.75187842	5.58027158
Up	ENSMUSG00000074151	2.365122161	2.11E-25	Nlrc5	8q	protein_coding	4.806471	5.041027	4.994276	7.011066275	7.28223557	7.1649493
Up	ENSMUSG00000027834	2.363000612	2.74E-03	Serpini1	3q	protein_coding	2.584619	2.998085	2.582785	4.468783477	4.08497815	3.80370098
Up	ENSMUSG00000066258	2.326393048	1.12E-24	Trim12a	7q	protein_coding	5.169009	4.95086	5.123489	7.109121719	7.48063841	7.1848469
Up	ENSMUSG00000071068	2.319721737	1.70E-16	Treml2	17q	protein_coding	4.458588	3.697788	4.242764	6.119286627	6.26370548	6.223974
Up	ENSMUSG00000049502	2.237224533	5.11E-24	Dtx3l	16q	protein_coding	5.458495	5.2445	5.16411	7.573423469	7.18666362	7.44621018
Up	ENSMUSG00000063268	2.210597844	2.20E-11	Parp10	15q	protein_coding	3.906135	4.166946	4.518158	6.076831664	6.06303335	6.33498506
Up	ENSMUSG00000096727	2.108555387	7.93E-32	Psmb9	17q	protein_coding	5.699488	6.062487	6.059937	7.881592075	7.96834533	8.09824426
Up	ENSMUSG00000030966	2.106299784	4.42E-06	Trim21	7q	protein_coding	2.806913	3.904082	3.802684	5.180082207	5.12640475	5.45477953
Up	ENSMUSG00000032265	2.088077368	1.11E-06	Tent5a	9q	protein_coding	3.584275	2.998085	3.695913	4.91679474	5.206555	5.0829478
Up	ENSMUSG00000029605	2.074849674	8.69E-03	Oas1b	5q	polymorphic_pseud	2.999485	3.167798	2.320622	4.096205315	3.99756313	4.38817558
Up	ENSMUSG00000071350	2.063410884	2.41E-08	Setdb2	14q	protein_coding	4.321104	3.997127	4.164838	5.817963911	6.33676059	5.63914819
Up	ENSMUSG00000026946	2.056224156	1.90E-24	Nmi	2q	protein_coding	5.671475	5.520064	5.6944	7.478770268	7.63343994	7.66740775
Up	ENSMUSG00000029366	1.954253216	1.55E-10	Dck	5q	protein_coding	4.699568	4.318864	4.579511	6.368418777	6.01931795	6.47084573
Up	ENSMUSG00000072620	1.921602784	1.15E-30	Slfn2	11q	protein_coding	7.032424	7.083744	6.947864	8.950908372	8.75833114	9.01172976
Up	ENSMUSG00000002307	1.881399513	3.66E-31	Daxx	17q	protein_coding	6.18885	6.018772	6.436701	8.145686207	7.86717026	8.1190767
Up	ENSMUSG00000090272	1.819725717	4.70E-45	Mndal	1q	protein_coding	7.043395	6.737779	7.377318	8.927207001	8.7748567	8.88974273
Up	ENSMUSG00000052749	1.786369786	6.23E-05	Trim30b	7q	protein_coding	3.999227	2.998085	3.695913	4.468783477	5.04153838	5.42162351
Up	ENSMUSG00000039997	1.753816892	7.34E-27	Ifi203	1q	protein_coding	6.168951	5.668895	6.102361	7.732305342	7.61886781	7.70233914
Up	ENSMUSG00000033538	1.748070421	8.51E-12	Casp4	9q	protein_coding	5.12837	4.90357	5.038645	6.470336243	6.76505364	6.68158151
Up	ENSMUSG00000022661	1.720833325	3.81E-06	Cd200	16q	protein_coding	4.458588	4.244903	4.316695	5.765466376	6.10546299	5.63914819
Up	ENSMUSG00000041736	1.719642268	4.85E-08	Tspo	15q	protein_coding	4.321104	4.318864	4.638361	5.817963911	6.08440415	5.94940894
Up	ENSMUSG00000027951	1.683963586	4.16E-11	Adar	3q	protein_coding	5.086553	4.751624	5.123489	6.550091248	6.58184799	6.51866674
Up	ENSMUSG00000027639	1.680681421	1.58E-26	Samhd1	2q	protein_coding	6.107553	5.903315	5.948076	7.534736457	7.551408	7.72289907
Up	ENSMUSG00000022867	1.615867942	7.73E-22	Usp25	16q	protein_coding	6.356571	6.97357	6.608434	8.25444563	8.41041602	8.05024141
Up	ENSMUSG00000045827	1.596729161	6.43E-05	Serpinb9	13q	protein_coding	4.64299	4.996648	4.518158	6.119286627	6.50468758	5.82813165
Up	ENSMUSG00000048895	1.513238161	2.15E-04	Cdk5r1	11q	protein_coding	4.699568	4.084534	4.749315	5.893293381	6.01931795	5.72318924
Up	ENSMUSG00000000275	1.477772287	2.43E-15	Trim25	11q	protein_coding	5.881682	6.125671	6.038248	7.42900821	7.31875936	7.55723281
Up	ENSMUSG00000028793	1.467033415	1.53E-08	Rnf19b	4q	protein_coding	5.953232	5.879072	5.242074	7.220567696	7.28223557	6.81524232
Up	ENSMUSG00000029780	1.361337709	2.81E-10	Nt5c3	6q	protein_coding	6.18885	5.996408	6.18364	7.462372891	7.35438128	7.48684891
Up	ENSMUSG00000023067	1.354726688	3.63E-07	Cdkn1a	17q	protein_coding	6.806361	7.625603	7.122927	8.268660962	8.7416141	8.61698575
Up	ENSMUSG00000059456	1.329678851	2.95E-13	Ptk2b	14q	protein_coding	6.128311	6.263154	5.775293	7.333065491	7.4480354	7.25240376
Up	ENSMUSG00000061731	1.282898556	1.32E-02	Ext1	15q	protein_coding	4.169123	4.389216	4.316695	5.295656743	5.42331732	5.45477953
Up	ENSMUSG00000026637	1.276952021	5.68E-08	Traf5	1q	protein_coding	4.999098	5.42279	5.242074	6.160527876	6.45632943	6.59500614
Up	ENSMUSG00000025746	1.267898866	7.76E-09	Il6	5q	protein_coding	8.032408	7.318192	7.768363	9.213468403	8.80091006	8.9167651

Up	ENSMUSG00000000184	1.267436713	1.01E-19	Ccnd2	6q	protein_coding	9.690718	9.673907	9.769919	10.99652977	11.0914934	10.8262004
Up	ENSMUSG00000006418	1.229155472	7.35E-12	Rnf114	2q	protein_coding	6.964786	6.353908	6.623078	7.881592075	7.69725221	8.00058623
Up	ENSMUSG00000026031	1.184446603	5.47E-16	Cflar	1q	protein_coding	7.293616	7.543145	7.297403	8.507150806	8.70068471	8.42120625
Up	ENSMUSG00000030530	1.182222029	1.82E-06	Furin	7q	protein_coding	5.553646	5.581451	5.548603	6.725233895	6.626238	6.66750827
Up	ENSMUSG00000028466	1.169348256	8.57E-02	Creb3	4q	protein_coding	4.169123	3.697788	3.318005	4.867832777	4.45677301	4.69611018
Up	ENSMUSG00000002728	1.124971992	2.29E-05	Naa20	2q	protein_coding	5.953232	5.829328	5.748829	6.905857143	6.85484311	6.98372804
Up	ENSMUSG00000066036	1.119754107	2.79E-10	Ubr4	4q	protein_coding	6.553602	6.878941	6.563588	7.697701445	7.82335574	7.76316028
Up	ENSMUSG00000042901	1.11766763	4.04E-07	Aida	1q	protein_coding	5.458495	5.455949	5.548603	6.58079835	6.52045334	6.50290187
Up	ENSMUSG00000039501	1.107132131	6.89E-05	Znfx1	2q	protein_coding	5.671475	5.31848	5.420331	6.403197414	6.52045334	6.60980098
Up	ENSMUSG00000060802	1.068393054	2.86E-34	B2m	2q	protein_coding	11.60167	11.59376	11.63732	12.6763065	12.6317898	12.7257795
Up	ENSMUSG00000019876	1.060908208	2.72E-06	Pkib	10q	protein_coding	5.831932	6.018772	6.059937	7.011066275	7.03027227	6.9139146
Up	ENSMUSG00000034575	1.03765701	6.22E-04	Tent4a	13q	protein_coding	5.490914	5.354095	5.123489	6.385912893	6.33676059	6.14491783
Up	ENSMUSG00000043279	1.035603816	3.44E-07	Trim56	5q	protein_coding	5.953232	6.225192	6.368812	7.077171389	7.38053215	7.08249551
Up	ENSMUSG00000020108	1.004939631	4.91E-02	Ddit4	10q	protein_coding	4.391483	3.997127	4.316695	5.139404073	5.28248582	4.80296907
Up	ENSMUSG00000066440	1.004018384	3.69E-02	Zfyve26	12q	protein_coding	4.52271	4.804071	4.638361	5.180082207	5.64086639	5.75014913
Down	ENSMUSG00000063065	-1.005618127	1.63E-03	Mapk3	7q	protein_coding	6.302802	6.816483	6.578692	5.595435969	5.77834625	5.51888961
Down	ENSMUSG00000031155	-1.015722458	3.11E-13	Pim2	Xq	protein_coding	9.389144	9.293109	9.432286	8.37322729	8.22561199	8.48679194
Down	ENSMUSG00000032009	-1.022324987	1.54E-05	Sesn3	9q	protein_coding	6.941517	6.581291	6.888494	5.682954614	5.69743991	6.10370354
Down	ENSMUSG00000025534	-1.03015807	1.31E-03	Gusb	5q	protein_coding	6.356571	6.371393	6.623078	5.367863493	5.24501989	5.80260298
Down	ENSMUSG00000000594	-1.031092083	7.15E-06	Gm2a	11q	protein_coding	6.086493	6.455774	6.637575	5.332211814	5.42331732	5.58027158
Down	ENSMUSG00000031112	-1.036883375	1.77E-02	Stk26	Xq	protein_coding	5.321	5.12589	5.386398	4.468783477	4.38968697	4.31783505
Down	ENSMUSG00000022051	-1.088397192	5.04E-02	Bnip3l	14q	protein_coding	4.391483	4.996648	4.518158	3.708356769	3.5827966	4.08355062
Down	ENSMUSG00000023990	-1.094731778	1.53E-02	Tfeb	17q	protein_coding	4.905997	5.084083	5.242074	4.096205315	4.08497815	4.31783505
Down	ENSMUSG00000024349	-1.132749672	7.92E-03	Sting1	18q	protein_coding	5.391385	5.16652	5.038645	4.331072792	3.90450792	4.45524542
Down	ENSMUSG00000020601	-1.169478352	1.60E-02	Trib2	12q	protein_coding	4.953299	5.31848	4.852342	3.915275444	4.1673978	4.16594581
Down	ENSMUSG00000057337	-1.173274547	4.12E-05	Chst3	10q	protein_coding	5.553646	6.125671	6.059937	4.964149438	5.04153838	4.58069866
Down	ENSMUSG00000021959	-1.197155603	2.06E-02	Lats2	14q	protein_coding	4.64299	4.640638	4.948498	3.328792046	3.90450792	4.08355062
Down	ENSMUSG00000038481	-1.254258099	1.58E-02	Cdk19	10q	protein_coding	5.043488	4.318864	4.694905	3.466710519	3.90450792	3.80370098
Down	ENSMUSG00000022186	-1.256359223	6.41E-05	Oxct1	15q	protein_coding	5.881682	5.950612	6.279195	5.009999048	4.90407428	4.80296907
Down	ENSMUSG00000026360	-1.280802396	7.93E-02	Rgs2	1q	protein_coding	4.458588	3.804619	3.902095	3.005722225	3.45736423	3.16708384
Down	ENSMUSG00000040061	-1.284787469	7.46E-02	Plcb2	2q	protein_coding	4.169123	4.084534	4.387022	3.17628163	3.31997893	3.45617673
Down	ENSMUSG00000026866	-1.28794643	8.11E-02	Kynu	2q	protein_coding	4.458588	4.244903	4.316695	3.466710519	3.45736423	3.3188594
Down	ENSMUSG00000005447	-1.328883308	2.33E-02	Pafah1b3	7q	protein_coding	4.699568	4.58177	4.638361	3.466710519	3.31997893	3.99616311
Down	ENSMUSG00000039981	-1.410794379	7.86E-03	Zc3h12d	10q	protein_coding	4.458588	5.084083	4.316695	2.812261074	3.99756313	3.69689834
Down	ENSMUSG00000039168	-1.423948243	9.26E-02	Dap	15q	protein_coding	4.169123	3.582409	4.082461	3.17628163	2.99837588	2.99744321
Down	ENSMUSG00000006517	-1.431833185	1.02E-02	Mvd	8q	protein_coding	5.208534	4.996648	5.123489	3.708356769	3.69819044	4.31783505
Down	ENSMUSG00000040451	-1.519139462	2.65E-04	Sgms1	19q	protein_coding	5.043488	5.16652	5.123489	3.328792046	3.99756313	4.16594581
Down	ENSMUSG00000003418	-1.589575759	1.19E-03	St8sia6	2q	protein_coding	5.831932	5.16652	6.059937	4.468783477	3.99756313	4.51933525
Down	ENSMUSG00000013846	-1.603778706	4.34E-03	St3gal1	15q	protein_coding	4.857092	4.244903	4.638361	3.708356769	2.99837588	3.45617673
Down	ENSMUSG00000074272	-1.665186084	4.61E-02	Ceacam1	7q	protein_coding	3.458776	4.084534	4.242764	2.588779838	2.80596293	3.3188594
Down	ENSMUSG00000049858	-1.676862734	2.96E-02	Suox	10q	protein_coding	4.64299	4.58177	5.31604	3.466710519	3.80503418	3.45617673
Down	ENSMUSG00000073678	-1.684807569	3.81E-05	Pgap1	1q	protein_coding	5.169009	5.084083	5.386398	3.815522598	3.90450792	3.80370098
Down	ENSMUSG00000051457	-1.71790737	6.35E-02	Spn	7q	protein_coding	3.906135	3.582409	4.164838	2.588779838	2.99837588	2.99744321
Down	ENSMUSG00000027984	-1.75490404	9.34E-04	Hadh	3q	protein_coding	5.12837	4.90357	5.123489	3.17628163	3.69819044	3.99616311
Down	ENSMUSG00000020573	-1.869448828	1.21E-05	Pik3cg	12q	protein_coding	4.905997	5.12589	5.517588	3.708356769	3.80503418	3.58155244
Down	ENSMUSG00000036880	-1.90150747	5.71E-02	Acaa2	18q	protein_coding	3.458776	3.997127	3.902095	3.005722225	2.80596293	2.32090592

Supplementary Table 2. Differentially expressed genes related to the metabolic process of CpG plus IFNα vs CpG
